# U3 snoRNA inter-regulates with DDX21 in the perichromosomal region to control mitosis

**DOI:** 10.1038/s41419-024-06725-3

**Published:** 2024-05-17

**Authors:** Yang Jiang, Shiqi Sun, Xiaofeng Liu, Kunqi Su, Chunfeng Zhang, Peipei Zhang, Zhuochen Zhao, Ya Su, Chang Wang, Xiaojuan Du

**Affiliations:** 1 Department of Cell Biology, School of Basic Medical Sciences, Peking University, Beijing, 100083 China; 2 Hepatopancreatobiliary Surgery Department I, Key laboratory of Carcinogenesis and Translational Research (Ministry of Education/Beijing), Cancer Hospital & Institute, Peking University, Beijing, 100142 China; 3 Department of Medical Genetics, School of Basic Medical Sciences, Peking University, Beijing, 100083 China; 4 Department of Biochemistry and Biophysics, School of Basic Medical Sciences, Peking University, Beijing, 100083 China

**Keywords:** Mitosis, Small RNAs

## Abstract

U3 snoRNA is essential for ribosome biogenesis during interphase. Upon mitotic onset, the nucleolus disassembles and U3 snoRNA relocates to the perichromosomal region (PR) to be considered as a chromosome passenger. Whether U3 controls mitosis remains unknown. Here, we demonstrate that U3 snoRNA is required for mitotic progression. We identified DDX21 as the predominant U3-binding protein during mitosis and confirmed that U3 snoRNA colocalizes with DDX21 in the PR. DDX21 knockdown induces mitotic catastrophe and similar mitotic defects caused by U3 snoRNA depletion. Interestingly, the uniform PR distribution of U3 snoRNA and DDX21 is interdependent. DDX21 functions in mitosis depending on its PR localization. Mechanistically, U3 snoRNA regulates DDX21 PR localization through maintaining its mobility. Moreover, Cy5-U3 snoRNA downsizes the fibrous condensates of His-DDX21 at proper molecular ratios in vitro. This work highlights the importance of the equilibrium between U3 snoRNA and DDX21 in PR formation and reveals the potential relationship between the PR assembly and mitotic regulation.

## Introduction

U3 snoRNA is a box C/D small nucleolar RNA that participates in the 18S rRNA processing through base-pairing with the 47S pre-rRNA at A_0_, A_1_ and A_2_ sites in the nucleolus [1]. U3 snoRNA carries out its functions in the ribosomal small subunit biogenesis in association with numerous U Three Proteins (UTPs). The UTPs form different subcomplexes that associate with the nascent pre-rRNA sequentially, including t-UTP/UTP-A, UTP-B and UTP-C subcomplexes which are required for pre-rRNA processing [2].

When cells enter prophase, rDNA transcription is repressed and the nucleolus structure disintegrates, resulting in the relocalization of nucleolar components including U3 snoRNP in the perichromosomal region (PR) [3, 4]. Early in 1992, immunoprecipitation assays showed that U3 snoRNA associates with perichromosomal protein antigens [5]. In 1994, U3 snoRNA was found to distribute in the PR during mitosis [6]. The PR is a ‘sheath’ coating the outer surface of the mitotic chromosomes and was originally found to consist of nucleolar components including U3 snoRNA [6], pre-rRNAs [7], fibrillarin, Ki-67 and nucleolin [8, 9]. In the past, the PR was supposed to be a binding site for chromosomal passenger proteins [10] or help to ensure the equal distribution of processing components between daughter cells [11]. U3 snoRNA has long been considered as a chromosome passenger [6], and if U3 snoRNA controls mitosis remains unknown. However, recent studies have demonstrated that several components of the PR are essential for mitotic progression, rather than serving as chromosome passengers. For instance, Ki-67, nucleolin and NOL11 are required for chromosome congression and segregation during mitosis [12–14]. These findings underline the unexpected roles of the PR in mitotic control. Importantly, the PR constitutes 30%-47% volume of the mitotic chromosomes and putative PR components comprise more than 33% of the mass of chromosomal proteins [15], suggesting that the potential functions of the PR are unexplored. Therefore, identification of the PR components and elucidation of the functions of the PR will help us to better understand the mechanisms of mitotic regulation.

The PR is a liquid-like membrane-less structure and is supposed to be assembled via promiscuous interactions among proteins and RNAs by liquid-liquid phase separation (LLPS) [16, 17]. RNAs are shown to promote phase separation by lowering the saturation concentration of numerous proteins [18]. Strong heterotypic interactions among proteins and RNA components stabilize the phase-separated condensates [19]. However, little is known about the functions of U3 snoRNA during PR assembly. We questioned whether U3 snoRNA facilitates phase separation of the PR-localized proteins.

A nucleolar RNA helicase DDX21 has been recently identified as a novel component of the UTP-B complex and plays multifaceted roles in ribosome biogenesis [20]. In the nucleolus, DDX21 participates in the Pol I transcription and rRNA processing by binding the transcribed rDNA region, pre-rRNA and snoRNAs, especially U3 snoRNA [21, 22]. DDX21 also regulates 2’-O-methylation and pseudouridylation of 18S, 5.8S and 28S rRNA [21]. Moreover, DDX21 facilitates the maturation of pre-40S particles through mediating the recruitment of late-acting snoRNAs to the pre-rRNA [20]. In the nucleoplasm. DDX21 promotes RNA Pol II elongation by facilitating the release of positive transcription elongation factor (P-TEFb) from 7SK snRNP to enhance the gene transcription of ribosomal proteins [21]. Recently, it has been found that the function of DDX21 in Pol I transcription is regulated by a long non-coding RNA *SLERT*, which regulates the liquidity of DDX21 and maintains the FC/DFC size [23]. Thus, the functions and distribution of DDX21 in the nucleolus are largely dependent on the interaction with RNA. During mitosis, DDX21 relocates to the PR [24, 25]. However, little is known about the role of DDX21 in mitotic regulation. Whether the PR location of DDX21 is regulated by RNA remains unclear.

In the present study, we found that U3 snoRNA is required for mitotic cell fate decision. We identified DDX21 as the predominant U3 snoRNA binding protein during mitosis. U3 snoRNA binds and co-localizes with DDX21 in the PR. Depletion of either U3 or DDX21 leads to mitotic defects and mitotic catastrophe. Importantly, the PR localization of U3 snoRNA and DDX21 is inter-dependent. DDX21 knockdown results in U3 snoRNA aggregation or absence on chromosomes. Depletion of U3 snoRNA causes DDX21 to form aggregate on chromosomes. Further, U3 snoRNA maintains the mobility of DDX21 in the PR and downsizes the gel-like aggregates of His-DDX21 at proper molecular ratios in vitro. In summary, our study provides novel insights into the functions of PR components in mitotic regulation.

## Results

### Depletion of U3 snoRNA leads to mitotic defects

**Fig. 1:**
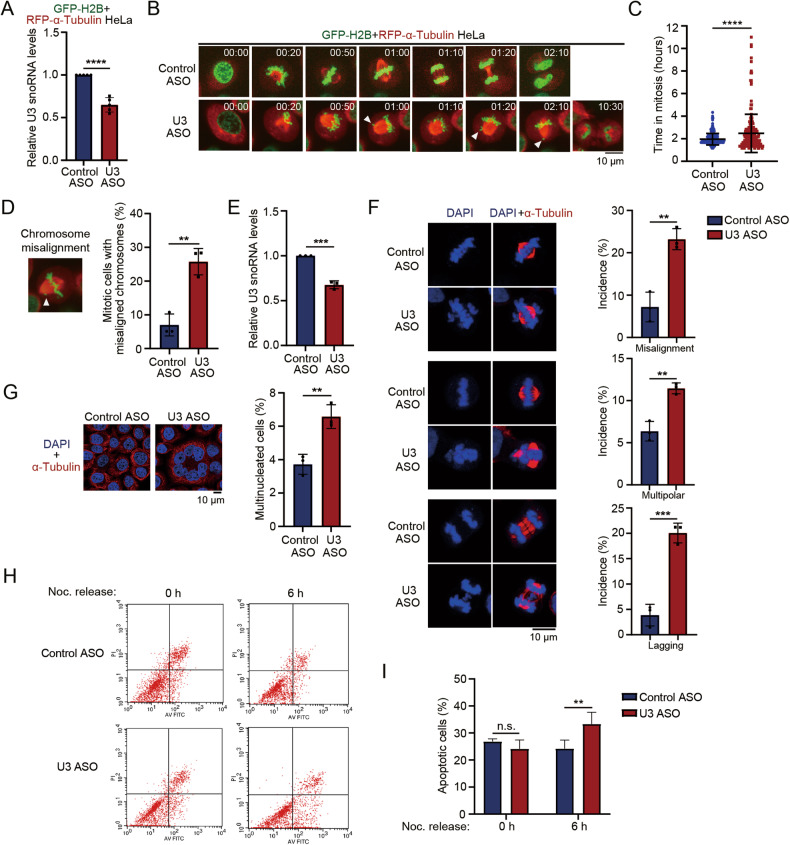
Depletion of U3 snoRNA leads to mitotic defects. **A** GFP-H2B + RFP-α-Tubulin HeLa cells were transfected with U3 snoRNA antisense oligonucleotide (ASO) or control ASO. U3 snoRNA level was evaluated by RT-qPCR. Data was summarized from five independent experiments. **B** Mitotic progression was monitored and recorded under time-lapse fluorescence microscopy in GFP-H2B + RFP-α-Tubulin HeLa cells at 48 h after transfection of ASOs as described in (**A**). Images were acquired every 10 min. Arrowheads point to the misaligned chromosomes in metaphase cells. Scale bar represents 10 μm. See Videos 1 and 2. **C** Time duration from nuclear envelope breakdown (NEBD) to mitotic exit was recorded in the cells described in (**B**). Control ASO: *n* = 221; U3 ASO: *n* = 224. Data is presented as means ± S.D. *P*-value was calculated using unpaired Student’s *t*-test. **D** Representative image of chromosome misalignment in the U3 snoRNA-depleted HeLa-GFP-H2B + RFP-α-Tubulin cells (left panel). A quantitative comparison of chromosome misalignment between control ASO and U3 ASO cells is shown. Data are representative of three independent experiments, error bars indicate S.D. **E** HeLa cells were transfected with the U3 snoRNA ASO or control ASO. Total RNAs were extracted, and RT-qPCR was performed to evaluate U3 snoRNA levels. **F** Indirect immunostaining was performed in cells described in (**E**) to visualize the mitotic spindle (α-Tubulin, red). Chromosomes were stained with DAPI. The frequency of chromosome misalignment, multipolar spindle and lagging chromosomes in the control ASO or U3 ASO cells is shown (n > 100). Data is summarized from three independent experiments. Scale bar, 10 μm. **G** Immunofluorescent staining was performed with anti-α-Tubulin antibody in HeLa cells transfected with indicated ASOs. Scale bar represents 10 μm. A quantitative comparison of multinucleated cells between HeLa cells transfected with control ASO or U3 ASO is shown. Data was summarized from three independent experiments (*n* > 500). **H** HeLa cells transfected with the indicated ASOs were synchronized to M phase using Thymidine-Nocodazole treatment. Mitotic cells were collected by mitotic shake-off and released into fresh medium for indicated times. Apoptotic cells were determined by flow cytometry. **I** Quantification of apoptotic cells is shown. *P*-values were calculated using two-tail unpaired Student’s *t-test*. **P < 0.01. ***P < 0.001. ****P < 0.0001. n.s. denotes no significance.

To determine if U3 snoRNA is required for mitosis, we knocked down U3 snoRNA by antisense oligonucleotides (ASOs) in the HeLa-GFP-H2B + RFP-α-Tubulin cells stably expressing GFP-H2B and RFP-α-Tubulin (Fig. 1A). Mitotic progression was recorded by time-lapse microscopy. Depletion of U3 snoRNA dramatically delayed the mitotic process (148.32 ± 101.82 min in the U3 snoRNA ASO cells *versus* 117.12 ± 30.636 min in the control ASO cells) (Fig. 1B, C; Videos 1 and 2). In addition, U3 snoRNA depletion increased chromosome misalignment in the metaphase (Fig. 1D). To define the knockdown of U3 snoRNA-caused mitotic defects, we transfected HeLa cells with U3 snoRNA ASO and visualized the chromosome and microtubule (Fig. 1E). Knockdown of U3 snoRNA leads to chromosome misalignment, multipolar spindle and lagging chromosomes (Fig. 1F). Consequently, depletion of U3 snoRNA leads to increased multinucleated cells (Fig. 1G), which is a hallmark of mitotic catastrophe. To further determine the U3 depletion-induced mitotic cell death, apoptotic cells were analyzed in M and the next G1 phase. We found that U3 depletion induced an increase of apoptotic cells in the next G1 phase (24.24 ± 3.17% control ASO vs 33.37 ± 4.33% U3 ASO cells), while no significant difference was found in M phase (Fig. 1H, I, S1A). These results indicate that U3 snoRNA is essential for mitotic cell fate decision.

### U3 snoRNA binds and colocalizes with DDX21 in the PR during mitosis

**Fig. 2:**
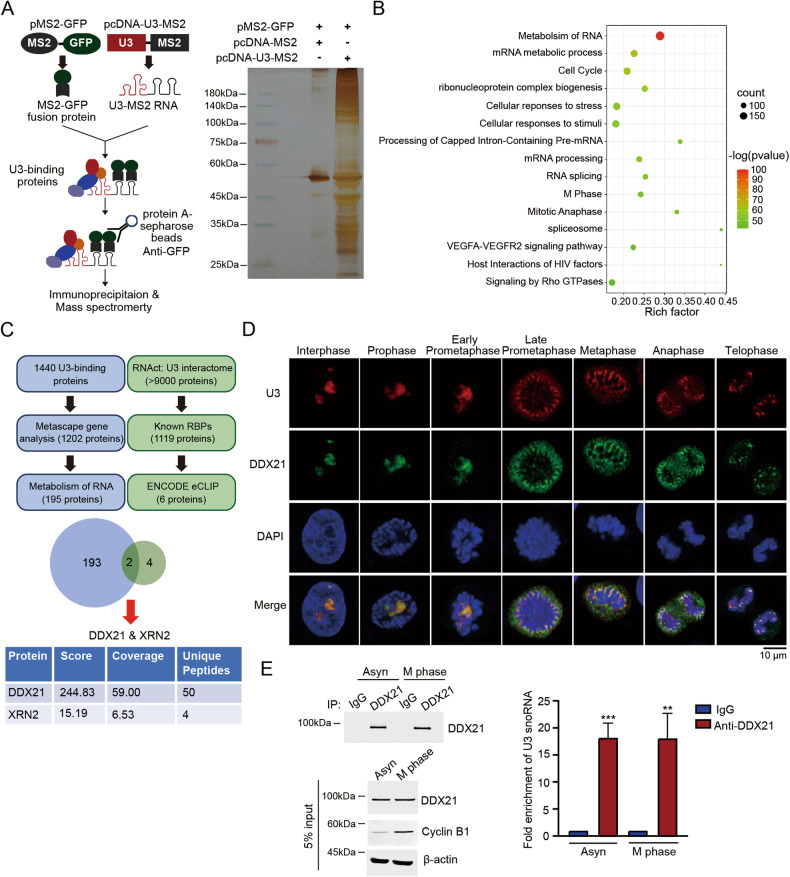
U3 snoRNA binds and colocalizes with DDX21 in the PR during mitosis. **A** Schematic illustration of MS2-pull down assay (left panel). HeLa cells were co-transfected with pcDNA-U3-MS2 and pMS2-GFP. Cells were synchronized at M phase by nocodazole (50 ng/ml) for 12 h and harvested. Whole-cell extracts were immunoprecipitated with anti-GFP antibody and the U3 snoRNA-binding proteins were resolved by SDS-PAGE, detected by silver staining and analyzed by mass spectrometry. **B** Enriched gene sets of U3 snoRNA-binding proteins are visualized by bubble plot. The size of each bubble represents the count of genes enriched in the total gene set. The color represents the *P*-value for the relevant pathway. **C** Flowchart of screening enrichment pathway of U3 snoRNA-binding proteins and prediction of U3 snoRNA-interacting RBPs. The Venn diagram shows overlapped proteins between the two groups. The table below shows the score, coverage and numbers of unique peptides of DDX21 and XRN2 from mass spectrometry. **D** Fluorescence in situ hybridization (FISH) was performed using a Cy3-labeled U3 snoRNA probe, and immunofluorescent staining was followed using an anti-DDX21 antibody to determine the endogenous localization of U3 snoRNA and DDX21 during the cell cycle. Scale bar, 10 μm. **E** HeLa cells were treated with 50 ng/ml nocodazole for 12 h to synchronize cells at M phase. RNA immunoprecipitation was performed in asynchronized and synchronized mitotic cells using anti-DDX21 antibody. DDX21 in the immunoprecipitants was evaluated by immunoblot. RNA was extracted and RT-qPCR was performed to detect U3 snoRNA in the immunoprecipitants. U3 snoRNA enrichment was determined relative to the non-targeting IgG control. *P*-values were calculated using unpaired *t*-test. **P < 0.01. ***P < 0.001.

To explore the mechanism by which U3 snoRNA functions in mitosis, we constructed pcDNA-U3-MS2 plasmid, performed an MS2-pull down experiment and mass spectrometry analysis to identify the U3 snoRNA-binding proteins in the mitotic cells (Fig. 2A). Thereafter, we comprehensively analyzed the gene set enrichment of U3 snoRNA-binding proteins (U3BP) using the Metascape website, a gene annotation and analysis resource. It shows that the most enriched pathway is the metabolism of RNA, including the processing of pre-mRNA, rRNA and tRNA, as well as mRNA capping, editing and decay (Fig. 2B, Table S1), in which 195 U3BPs have been found. We therefore analyzed the RNA binding proteins (RBPs) in the U3 snoRNA interactome. The RNAct database [26] shows that more than 9 000 proteins are predicted as the U3 snoRNA interactome, in which 1 119 proteins are known as RBPs. Six out of these 1119 RBPs have been confirmed to interact with U3 snoRNA by the ENCODE eCLIP project (Table S1) [27]. By intersecting these 6 RBPs with the 195 U3BPs which are involved in the metabolism of RNA in mitotic cells, we defined two overlapping proteins, DDX21 and XRN2 (Fig. 2C). Since the score and coverage of DDX21 in mass spectrometry are much higher than that of XRN2 (Fig. 2C), we identified DDX21 as the predominant U3BP during mitosis. In order to obtain an overall image of the spatial localization of U3 snoRNA and DDX21 in mitotic cells, we performed RNA fluorescence in situ hybridization (FISH) with a U3 snoRNA probe and immunofluorescent staining with anti-DDX21 antibody. As shown in Fig. 2D, U3 snoRNA co-localizes with DDX21 in the nucleoli during interphase and prophase, while they co-localize in the PR from late prometaphase to anaphase. Both U3 snoRNA and DDX21 distribute uniformly and continuously in the PR. During mitotic exit, both U3 snoRNA and DDX21 accumulate to the pre-nucleolar bodies (PNBs) as previously reported [25, 28]. We further confirmed the binding between U3 snoRNA and DDX21 by RNA immunoprecipitation (RIP) experiment in the asynchronized cells and mitotic cells, respectively (Fig. 2E). These results indicate that U3 snoRNA binds with DDX21 from the interphase to mitosis, suggesting that U3 snoRNA might collaborate with DDX21 to control mitosis, and the unique PR localization of U3 snoRNA and DDX21 might contribute to their functions in mitotic cell fate decision.

### DDX21 depletion results in mitotic defects and mitotic catastrophe

**Fig. 3:**
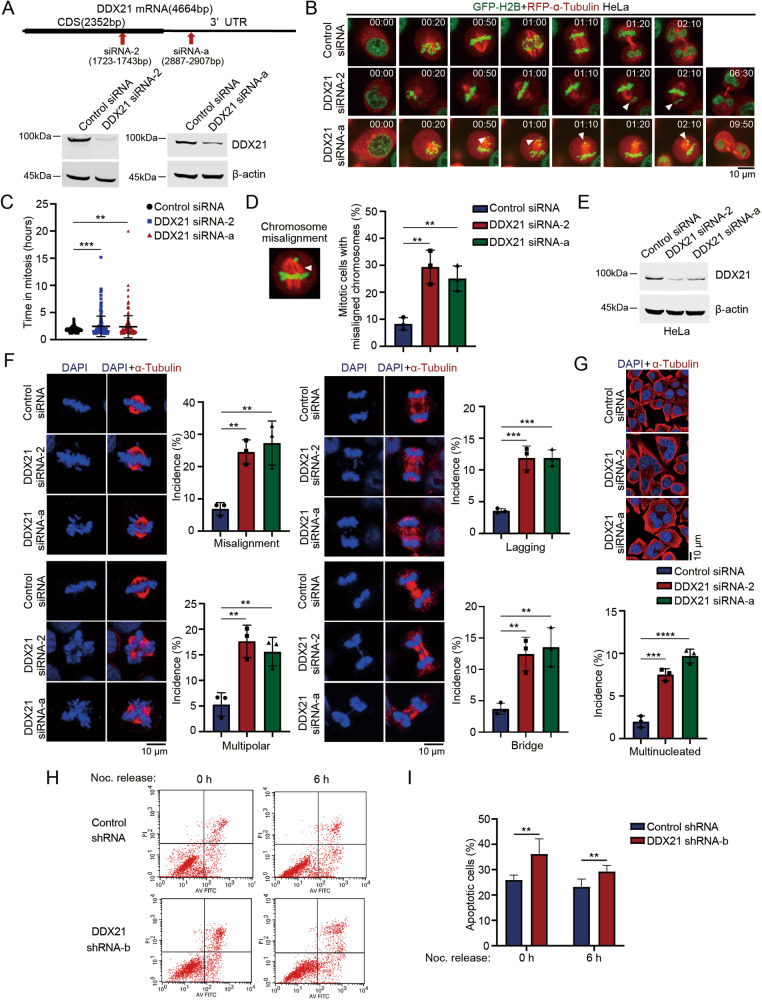
DDX21 depletion results in mitotic defects and mitotic catastrophe. **A** The target sites of siRNAs are shown in the schematic diagram (upper panel). GFP-H2B + RFP-α-Tubulin HeLa cells were transfected with siRNAs targeting the CDS region and 3’UTR region of DDX21 mRNA, respectively. Whole-cell extracts were immunoblotted to evaluate DDX21 protein levels. **B** Mitotic process of GFP-H2B + RFP-α-Tubulin HeLa cells described in (**A**) was monitored by time-lapse fluorescence microscopy for 24 h and snapshots were taken at indicated time points. Arrowheads point to the chromosomes that failed to congress at the metaphase plate. Scale bar represents 10 μm. See Videos 3, 4 and 5. **C** Mitotic time was recorded in the cells described in (**B**). Control siRNA: n = 192; DDX21 siRNA-2: n = 193; DDX21 siRNA-a: *n* = 183. Data is presented as means ± S.D. *P*-values were calculated using one-way ANOVA. **D** Chromosomal misalignment in the DDX21-depleted GFP-H2B + RFP-α-Tubulin HeLa cells is shown by snapshot (left panel). A summary of the frequency of chromosomal misalignment in DDX21-depleted cells compared to control cells is shown (right panel). Data were analyzed by one-way ANOVA. **E** HeLa cells were transfected with indicated siRNAs. Whole-cell extracts were extracted, and Western blotting was performed to evaluate DDX21 protein levels. Beta-actin was used as a loading control. **F** HeLa cells described in (**E**) were fixed, and immunofluorescent staining was performed using anti-α-tubulin antibody. Chromosomes were stained with DAPI. Scale bar represents 10 μm. The percentage of chromosomal misalignment, multipolar spindle, chromosomal bridge and lagged chromosomes is presented (nå 100). Data were summarized from three independent experiments. **G** Cells described in (**E**) were immunostained with anti-α-tubulin antibody. A quantitative comparison of multinucleated cells (n > 500) between control cells and DDX21 knockdown cells is presented. Scale bar, 10 μm. Data were summarized from three independent experiments. *P*-values were calculated using one-way ANOVA. *P < 0.05. **P < 0.01. ***P < 0.001. ****P < 0.0001. **H** Control shRNA and DDX21 shRNA-b HeLa cells were synchronized to M phase using Thymidine-Nocodazole treatment. Mitotic cells were collected by mitotic shake-off and released into fresh medium for indicated times. Apoptotic cells were determined by flow cytometry. **I** Quantification of apoptotic cells is shown. **P < 0.01.

To determine if DDX21 is also indispensable for mitosis, we silenced DDX21 in HeLa-GFP-H2B + RFP-α-Tubulin cells by siRNA (Fig. 3A) and monitored mitotic progression by time-lapse microscopy. The mitosis process was dramatically delayed when DDX21 was depleted (147.48 ± 113.16 min in siRNA-2 cells; 142.62 ± 121.38 min in siRNA-a cells *versus* 109.92 ± 27.15 min in control siRNA cells) (Fig. 3B, C; Videos 3–5). In addition, DDX21 depletion leads to chromosome misalignment in the metaphase (Fig. 3D). To further explore the mitotic defects induced by DDX21 depletion, we silenced DDX21 in HeLa cells (Fig. 3E). Depletion of DDX21 resulted in chromosome misalignment, multipolar spindle, lagging chromosomes and chromatin bridge (Fig. 3F). As a consequence, depletion of DDX21 leads to mitotic catastrophe, represented by increased multinucleated cells (Fig. 3G). To further examine the mitotic-induced apoptosis after DDX21 knockdown, we constructed a DDX21 knockdown stable cell line (Fig. S2A). We found that DDX21 depletion induces an increase of apoptotic cells during mitosis (25.95 ± 1.919% control shRNA vs 36.19 ± 5.975% DDX21 shRNA-b cells) and in the next G1 phase (23.22 ± 3.058% control shRNA vs 29.27 ± 2.362% DDX21 shRNA-b cells) (Fig. 3H, I, S2B). These results indicated that both U3 snoRNA and DDX21 are essential for mitotic control and loss of either U3 snoRNA or DDX21 causes mitotic catastrophe. Moreover, DDX21 depletion induced apoptosis earlier than U3 snoRNA depletion did.

### U3 snoRNA and DDX21 inter-regulate each other in the PR

**Fig. 4:**
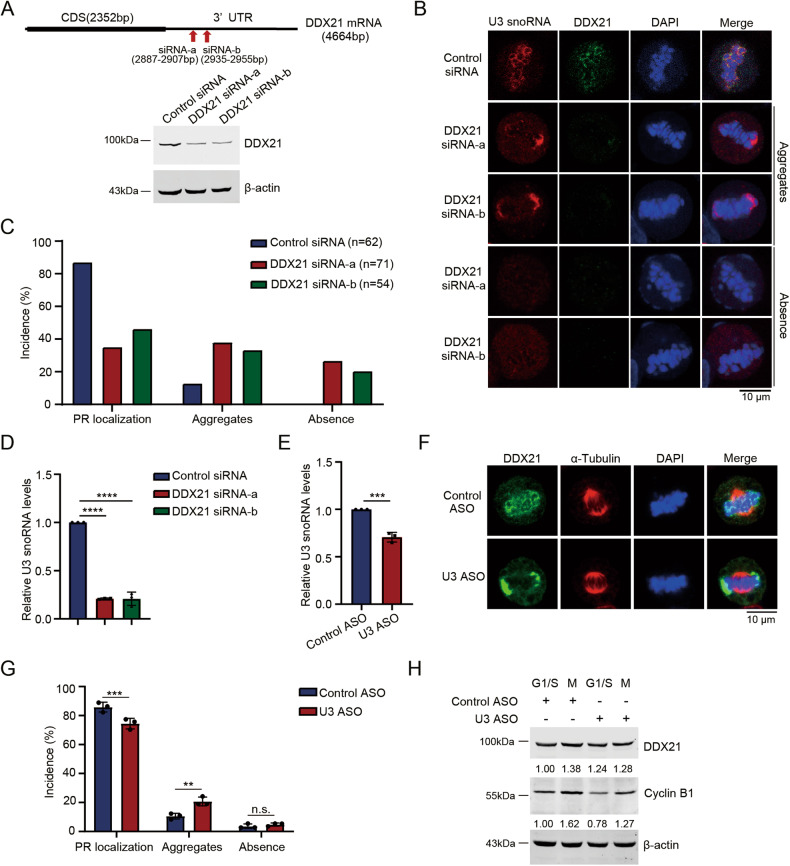
U3 snoRNA and DDX21 inter-regulate each other in the PR. **A** HeLa cells were transfected with either DDX21 siRNA-a or DDX21 siRNA-b as illustrated in the upper panel. HeLa cells were harvested, and Western blotting was performed to evaluate DDX21 protein levels. Beta-actin was used as a loading control. **B** The cells described in (**A**) were fixed and stained for endogenous U3 snoRNA using FISH followed by immunofluorescent staining of DDX21. Representative snapshots of HeLa cells in metaphase are shown. Scale bar, 10 μm. **C** The incidences of localization patterns of U3 snoRNA in the cells described in (**B**) are summarized. **D** Total RNAs were extracted from the HeLa cells described in (**A**) and RT-qPCR was performed for evaluating U3 snoRNA levels. Data was analyzed using one-way ANOVA. ****P < 0.0001. **E** HeLa cells were transfected with U3 ASO or control ASO. U3 snoRNA level was evaluated by RT-qPCR. **F** HeLa cells described in (**E**) were fixed and indirect immunofluorescent staining was performed using anti-α-tubulin and anti-DDX21 antibodies. Chromosomes were stained by DAPI. Scale bar, 10 μm. **G** The incidences of each pattern of DDX21 localization are shown. Data was summarized from three independent experiments. *P*-values were calculated using Student’s unpaired *t-test*. **P < 0.01. ***P < 0.001. n.s. denotes no significance. **H** HeLa cells described in (**E**) were synchronized by thymidine block for G1/S arrest or nocodazole treatment for arrest in M phase. After synchronization, cells were harvested, and Western blotting was performed to evaluate the levels of indicated proteins.

The PR is supposed to be a phase-separated organelle [29], and protein-RNA interactions drive the assembly of these membrane-less structures [18]. Since U3 snoRNA and DDX21 colocalize in the PR, we wondered if DDX21 and U3 snoRNA regulate each other to maintain their PR localization. We first examined if the localization of U3 snoRNA is affected by DDX21 during mitosis. When DDX21 was silenced, U3 snoRNA exhibited two distinct abnormal localization patterns: 1) U3 snoRNA forms aggregates on chromosomes in 38.03% of HeLa-DDX21 siRNA-a cells; 33.33% of HeLa-DDX21 siRNA-b cells *versus* that in 12.9% of HeLa-control siRNA cells (Fig. 4A–C); 2) U3 diminishes in 26.76% of HeLa-DDX21 siRNA-a and 20.37% of HeLa-DDX21 siRNA-b cells (Fig. 4B, C). We therefore evaluated the level of U3 snoRNA and found that DDX21 depletion caused downregulation of U3 snoRNA (Fig. 4D), which could explain the absence of U3 snoRNA in some DDX21-depleted cells. We further verified the aberrant distribution of U3 snoRNA in DDX21-knockdown stable cell lines (Fig. S3A). U3 snoRNA displayed similar aggregates on chromosomes in 29.27% of HeLa-DDX21-shRNA-a and 25.81% of HeLa-DDX21-shRNA-b cells; U3 snoRNA showed weak signal on chromosomes in 20.73% of DDX21-shRNA-a and 37.1% of DDX21-shRNA-b cells (Fig. S3B, C). In contrast, DDX21 forms aggregates on chromosomes when U3 snoRNA was depleted (Fig. 4E–G, Fig. S4A), while DDX21 protein levels were not affected (Fig. 4H). Together, we showed that the PR residence of U3 snoRNA and DDX21 are inter-dependent. These data strongly imply that the liquidity and mobility of U3 snoRNA and DDX21 in the PR are regulated by the molecular ratios between U3 snoRNA and DDX21.

### DDX21 controls mitosis dependent on the PR localization and its functional domains

**Fig. 5:**
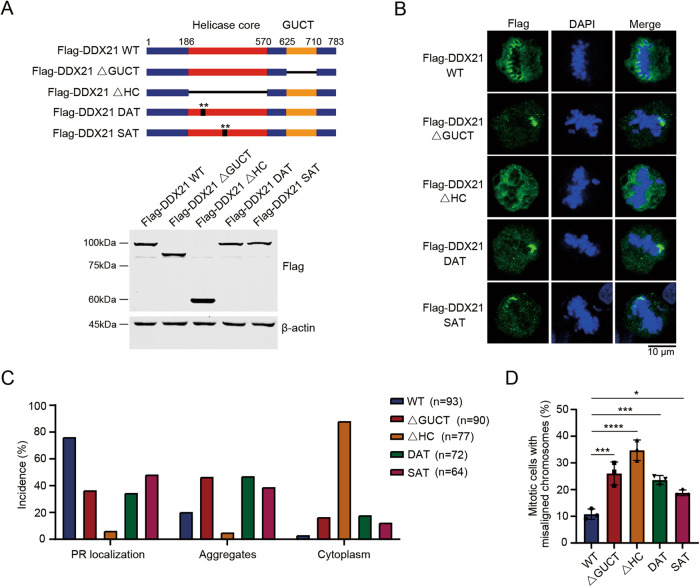
DDX21 controls mitosis dependent on the PR localization and its functional domains. **A** HeLa cells were transfected with Flag-DDX21^WT^ or different Flag-DDX21 mutants. Schematic diagram represents the constructs of Flag-DDX21 mutants (upper panel). Cell lysates were extracted, and proteins were subjected to Western blots probed with anti-Flag antibody (lower panel). **B** Immunofluorescent staining was performed using anti-Flag antibody. Chromosomes were stained with DAPI. Localization of Flag-DDX21^WT^ and Flag-DDX21 mutants during metaphase was visualized by confocal fluorescence microscopy. Scale bar, 10 μm. **C** The incidences of each localization pattern of Flag-DDX21 in HeLa cells were summarized. **D** The incidences of mitotic HeLa cells with chromosomal misalignment were presented. Data was summarized from three independent experiments. *P*-values were calculated using one-way ANOVA. *P < 0.05. ***P < 0.001. ****P < 0.0001.

DDX21 plays multifaceted roles in maintaining cellular homeostasis, relying on its multiple functional domains including the helicase core (HC) and a Gu C-terminal (GUCT) domain [30]. Additionally, the ATP-binding motif and ATP-hydrolysis motif are included in the HC domain [31]. We further questioned which functional domain or motif determines the distribution of DDX21 in the PR. Therefore, we constructed Flag-DDX21 truncation plasmids, as well as Flag-DDX21^DAT^ and Flag-DDX21^SAT^ mutants, in which ATP-binding and ATPase activity was abolished, respectively [31, 32] (Fig. 5A). The cellular localization patterns of Flag-DDX21 and its mutants in mitotic cells were determined. Flag-DDX21^WT^ mainly localized in the PR. However, Flag-DDX21△HC lost PR localization and dispersed in the cytoplasm, suggesting that the helicase core domain is essential for its localization in the PR (Fig. 5B). In contrast, Flag-DDX21△GUCT, Flag-DDX21^DAT^ and Flag-DDX21^SAT^ partially displayed as aggregates in the PR (Fig. 5B, C), similar to the aberrant distribution of DDX21 when U3 was depleted. These results suggested that the RNA-recognition, ATP-binding and ATPase motifs are required for its regular distribution in the PR. Unexpectedly, ectopically expressed Flag-DDX21^WT^ also partially presents as irregular aggregates on chromosomes, and U3 snoRNA levels were not changed after ectopic expression of Flag-DDX21^WT^ (Fig. S5A, B). Based on these observations, we presumed that over-abundant exogenous Flag-DDX21 tends to form aggregates due to the molecular imbalance between U3 snoRNA and DDX21. These results further confirm that the proper PR distribution depends on the molecular ratio between U3 and DDX21. Importantly, we observed an increase of chromosome misalignment in HeLa cells when these Flag-DDX21 mutants were ectopically expressed (Fig. 5D), which indicates that DDX21 controls mitosis dependent on its PR localization and functional domains.

To determine if the ectopic expression of Flag-DDX21 could rescue DDX21 depletion-induced multinucleation, we ectopically expressed the Flag-DDX21 mutants into the DDX21-KD stable cell line (Fig. S6A). However, the reintroduction of Flag-DDX21^WT^ in the DDX21 shRNA-b cell line did not significantly change the phenotype of multinucleated cells, indicating that multinucleation caused by DDX21 depletion is irreversible. In contrast, ectopic expression of Flag-DDX21 mutants in the DDX21-depleted cells significantly increased the percentage of multinucleated cells (Fig. S6B, C), implying that the PR residence of DDX21 is essential for the mitotic control. This is further verified by the fact that ectopic expression of Flag-DDX21 mutants in the control shRNA cells also slightly increased the portion of multinucleated cells (Fig. S6B, C)). These results further demonstrate that the conserved domains and motifs of DDX21 are required for the PR distribution and mitotic cell fate decision.

### U3 snoRNA maintains the mobility of DDX21 in the PR

**Fig. 6:**
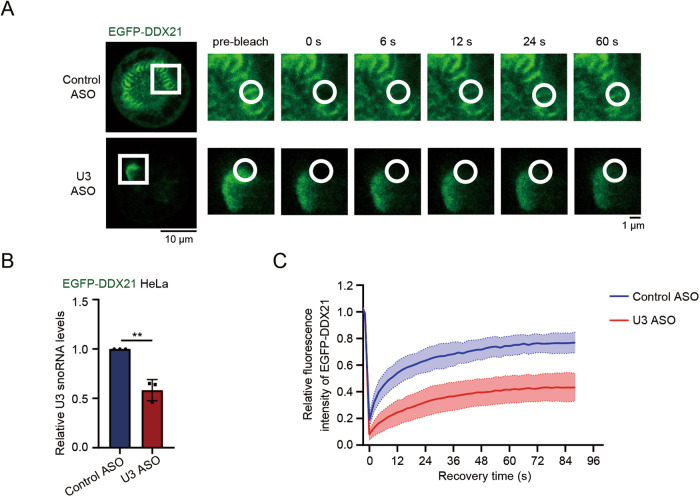
U3 snoRNA maintains the mobility of DDX21 in the PR. **A** HeLa cells stably expressing EGFP-DDX21 were transfected with U3 ASO or control ASO. After 48 h, FRAP analyses were performed. Left panel: representative snapshots of EGFP-DDX21 in mitotic cells; scale bar, 10 μm. Right panel: magnified views of the region in the white box at different time points (s); the white circles label the regions that were bleached; scale bar, 1 μm. **B** U3 snoRNA levels of cells described in (**A**) were evaluated by RT-qPCR. *P*-values were calculated using Student’s unpaired *t-test*. **P < 0.01. **C** Quantification of the relative fluorescence intensity of EGFP-DDX21 over time in FRAP experiments. Data were presented as mean ± SD, *n* > 30.

To further determine the functions of U3 snoRNA in PR, we identified whether U3 snoRNA regulates the mobility of DDX21 in the PR. We performed fluorescence recovery after photobleaching (FRAP) assay in HeLa cells stably expressing EGFP-DDX21. PR-localized EGFP-DDX21 showed rapid and nearly 75% recovery of fluorescence intensity after photobleaching in control cells (Fig. 6A–C), demonstrating that DDX21 is highly mobile in the PR. However, EGFP-DDX21 formed irregular aggregates on the chromosomes and showed significantly lower recovery (~40%) of fluorescence intensity in the U3 snoRNA knocked-down cells (Fig. 6A–C), illustrating that the homogenous PR localization and mobility of DDX21 are controlled by U3 snoRNA. Taken together, our data suggest that DDX21 might undergo a phase transition from a liquid-like PR localization pattern to aggregates on the chromosomes after U3 snoRNA knockdown.

### U3 snoRNA downsizes the gel-like fiber of DDX21 at a range of molecular ratios in vitro

**Fig. 7:**
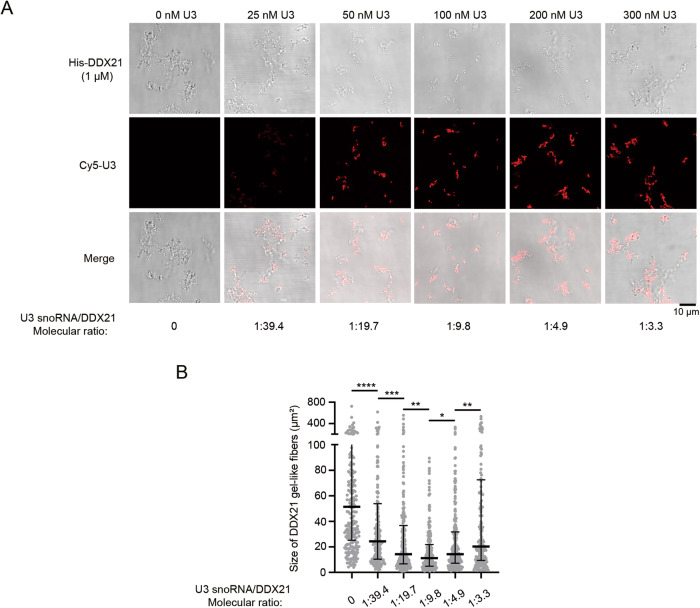
U3 snoRNA downsizes the gel-like fiber of DDX21 in a range of molecular ratios in vitro. **A** Droplet formation assay was performed with His-DDX21 (1 μM). Cy5-U3 snoRNA was added at increasing concentrations (0–300 nM) to the purified His-DDX21 (1 μM) and incubated with droplet assay buffer. Representative images of gel-like fibers were shown. Scale bar represents 10 μm. **B** Statistics of the size of DDX21 fibers in (**A**) were shown (*n* > 200). Data were presented as medians + upper quartiles and medians – lower quartiles. *P*-values were calculated using Kruskal-Wallis test. *P < 0.05. **P < 0.01. ***P < 0.001. ****P < 0.0001.

To further explore the effect of U3 snoRNA on the mobility of DDX21 in vitro, we purified His-DDX21 (Fig. S7A) and performed in vitro droplet formation assays. At near-physiological salt concentration (100 mM NaCl), we found that 1 μM His-DDX21 forms amorphous gel-like aggregates in vitro (Fig. 7A) as previously reported [32]. We also noticed that the DDX21 filamentous structures were resistant to hexanediol (HEX) treatment (Fig. S7B), which is widely used to dissolve phase-separated condensates, suggesting that DDX21 fibers lack liquidity in vitro. We next added Cy5-U3 snoRNA to the purified His-DDX21 in different concentrations. Mixing of Cy5-U3 snoRNA with His-DDX21 led to the formation of co-localized filamentous aggregates in vitro (Fig. 7A). Notably, the addition of 25–300 nM U3 snoRNA to 1 μM DDX21 modifies the size and morphology of DDX21 aggregates in a dose-dependent way. DDX21 forms minimal-sized condensates at a concentration of 1 μM with 100 nM U3 snoRNA, in which the molecular ratio of U3 snoRNA/DDX21 is about 1:9.8 (Fig. 7B). Importantly, the U3 snoRNA/DDX21 condensates appear as branched networks of droplets under this molecular ratio (Fig. 7A), suggesting that U3 snoRNA might facilitate the homogenous distribution and mobility of DDX21 in the PR by downsizing the DDX21 gel-like fibers at a range of molecular ratios.

### DDX21 and U3 snoRNA localize in the PR dependent on the presence of pre-rRNA

**Fig. 8:**
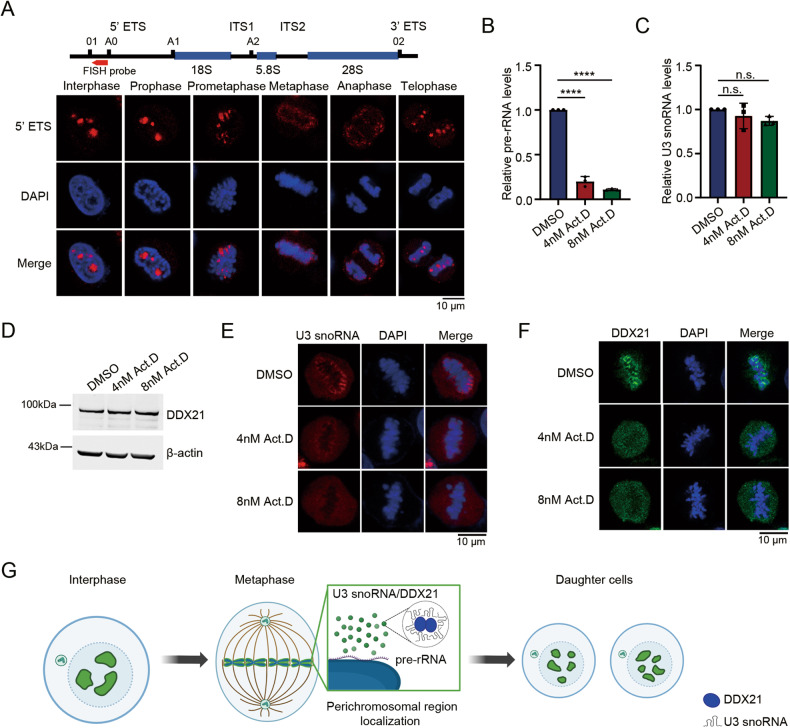
DDX21 and U3 snoRNA localize in the PR dependent on the presence of pre-rRNA. **A** FISH was performed using a Cy3-labeled 5’ ETS pre-rRNA probe in HeLa cells. Scale bar = 10 μm. The position of the Cy3-labeled FISH probe in the 5’ ETS of 47S pre-rRNA is shown in the upper panel. **B** HeLa cells were treated by actinomycin D (4 nM, 8 nM) or DMSO for eight hours. Total RNAs were extracted, and RT-qPCR was performed to evaluate pre-rRNA levels. Data was analyzed using one-way ANOVA. ****P < 0.0001. **C** Total RNAs were extracted from cells described in (**B**). RT-qPCR was performed using U3 snoRNA primers. *P*-values were calculated by one-way ANOVA. n.s. denotes no significance. **D** HeLa cells were treated with actinomycin D or DMSO for 8 h. Cells were synchronized at M phase by the addition of 50 ng/ml nocodazole for another 12 h. Whole-cell lysates were prepared and immunoblotted using anti-DDX21 antibody. **E** U3 snoRNA FISH was performed using a Cy3-labeled U3 probe in HeLa cells described in (**B**). Representative snapshots of metaphase HeLa cells are shown. **F** After actinomycin D treatment, HeLa cells were fixed and immunostained with anti-DDX21 antibody. Images of metaphase HeLa cells were acquired by confocal microscopy. **G** A working model illustrating that mitotic cell fate decision regulated by U3-DDX21 interplay in the PR. The figure was created with BioRender.com.

We next asked by what mechanism U3 and DDX21 are tethered to the PR. During interphase, DDX21 localizes in the nucleolus dependent on the RNA Pol I activity [21], which is responsible for pre-rRNA transcription. Since pre-rRNA particles were found to localize in the PR [8], we proposed that the PR distribution of DDX21 might rely on the presence of pre-rRNAs. As previously described [3], the FISH images showed the localization of pre-rRNAs in the PR during mitosis (Fig. 8A). When the pre-rRNA transcription was specifically suppressed by low-dose actinomycin D treatment (Fig. 8B) as described previously [33], U3 snoRNA and DDX21 lost their PR localization and dispersed in the cytoplasm (Fig. 8E, F), whereas U3 snoRNA and DDX21 levels were not affected (Fig. 8C, D). In addition, we noticed that neither U3 snoRNA nor DDX21 formed aggregates on the chromosomes, suggesting that they might form minimal and homogeneous condensates in cells as the endogenous molecular ratio of U3 snoRNA/DDX21 was not changed. These results indicated that the PR localization of DDX21 and U3 snoRNA depends on the presence of pre-rRNA. Taken together, we demonstrate that U3 snoRNA and DDX21 regulate each other in the PR to control mitotic cell fate (Fig. 8G).

## Discussion

U3 snoRNA works as an RNA chaperone within the small subunit (SSU) processome, mediating rRNA folding, modifications, rearrangements and cleavage to participate in the pre-rRNA processing in the interphase [34, 35]. Upon the onset of mitosis, U3 snoRNA relocates to the PR [6]. However, little is known about the functions of U3 in mitosis. In the present study, we found that U3 snoRNA depletion causes mitotic defects and leads to mitotic catastrophe, indicating that U3 snoRNA plays a critical role in determining mitotic cell fate, rather than serving as a chromosomal passenger.

Given that the PR is one of the membrane-less organelles consisting of RNA and proteins, we speculate that U3 snoRNA acts in mitosis depending on the association with proteins in the PR. We therefore set out to explore the U3BPs that play essential roles in the U3 snoRNA-mediated mitotic control. We performed MS2 pull-down experiment to identify the U3BPs in mitotic cells and analyzed the enriched pathways of gene sets. The results indicated that the metabolism of RNA is the most enriched pathway among U3BPs, which includes 195 proteins. To define the critical U3-binding RBPs acting in the U3 snoRNA-mediated mitotic control, we screened the U3 snoRNA interactome using the RNAct database. Among the 1119 predicted U3-binding RBPs, 6 proteins have been confirmed to associate with U3 snoRNA in the ENCODE eCLIP project [27]. After conducting a cross-analysis of these 6 U3-binding RBPs with the 195 U3BPs enriched in the RNA metabolism pathway, we found that DDX21 and XRN2 are the overlapped proteins. Although XRN2 functions in pre-rRNA maturation and decay [36], it mainly localizes in the nucleoplasm, implying that the interaction of U3 snoRNA with XRN2 is much weaker than that with DDX21. Besides, the score of DDX21 in mass spectrometry analysis of U3BPs in mitotic cells is much higher than that of XRN2. It was previously found that U3 snoRNA is the most enriched short repetitive RNA in the DDX21-bound RNAs [21]. Therefore, DDX21 was identified as the predominant U3BP during mitosis. We show that DDX21 depletion photocopied the U3 knockdown-induced mitotic catastrophe and mitotic defects, leading to cell death, suggesting that these two PR components might collaborate to determine mitotic cell fate. We further explored the mechanisms by which U3 snoRNA and DDX21 regulate mitosis. We performed quantitative mass spectrum analysis in DDX21 shRNA-b and control shRNA HeLa cells, as well as U3 ASO and control ASO HeLa cells, and screened proteins with significant differences in expression levels. We analyzed the gene set enrichment of these proteins using Metascape website and focused on the proteins involved in the “Mitotic cell cycle” gene set. By intersecting these proteins with 82 mitotic-related genes in the gene set GO:0007067 from Gene Ontology, 7 downregulated proteins after DDX21 knockdown and 4 upregulated proteins after U3 snoRNA depletion were identified (Fig. S8A–C). These proteins are responsible for spindle assembly or for chromosome condensation, suggesting that U3 snoRNA and DDX21 might control mitosis through regulating these genes. However, the specific mechanisms need further study.

Although technological advances have facilitated the exploration of novel components of PR [37, 38], the mechanisms underlying the assembly of this membrane-less compartment remain largely elusive. We observed that U3 snoRNA and DDX21 both distribute uniformly and continuously in the PR as reported [6, 24]. Interestingly, DDX21 forms aggregates on the chromosomes in U3-knockdown cells, and U3 forms similar aggregates in a portion of DDX21-depleted cells, indicating that the balance between U3 and DDX21 maintains their proper PR distribution. Considering that the assembly of the PR might be controlled by LLPS, the uneven distribution of DDX21 in the PR aroused our special interest and we wondered if U3 regulates the aggregation of DDX21 via LLPS. We showed that DDX21 exhibits rapid molecular dynamics in the PR as seen in FRAP analyses, confirming that the PR is liquid-like. However, in the U3-depleted cells, DDX21 aggregates on the chromosomes with slower fluorescence recovery rate, demonstrating that the uneven distribution of DDX21 is due to its poor mobility. Our data indicate that U3 snoRNA maintains the liquidity of DDX21 in the PR. In agreement with recently published data [32], we further found that His-DDX21 itself forms large filamentous condensates in vitro. Cy5-U3 snoRNA decreases the size and partially changes the morphology of DDX21 aggregates at a range of molecular ratios. His-DDX21 assembles into downsized branched networks of liquid-like beads when U3 snoRNA/DDX21 molecular ratio was around 1:9.8, while larger filamentous condensates with less liquid-like appearance were generated when the molecular ratios were out of the range. Similar morphological changes of the aggregates were reported previously [39]. These results demonstrate that U3 snoRNA might partially augment the liquidity of DDX21 aggregates and initiate the gel-to-liquid phase transition of DDX21 in the PR. RNAs have been shown to drive the assembly of condensates through mediating protein conformational switches and regulating electrostatic forces [40, 41]. Importantly, it was found that DDX21 conformation and liquidity are regulated by lncRNA *SLERT* through a molecular chaperone-like mechanism [32], providing strong evidence that the LLPS of DDX21 might be regulated by RNAs. We thus speculate that U3 snoRNA might act as a chaperone to maintain the mobility of DDX21 in the PR during mitosis.

Since His-DDX21 failed to form liquid-like spherical droplets in vitro, even in the presence of U3 snoRNA, we propose that there might be other proteins and RNAs augmenting the mobility and liquidity of DDX21 in the PR. We further evaluated the ability of DDX21 to drive droplet formation using the FuzDrop web server (fuzdrop.bio.unipd.it/predictor) [42]. The probability of spontaneous LLPS (*p*_LLPS_) of DDX21 is 0.4512, lower than the threshold (*p*_LLPS_ = 0.60), confirming that DDX21 could not form droplets spontaneously. Therefore, DDX21 could be defined as a droplet-client protein that requires interactions with partners or specific cellular conditions to form liquid-like droplets. A PR-localized protein fibrillarin was shown to generate spherical liquid-like droplets with DDX21 in a range of molar ratios in vitro [32], we thus wondered if fibrillarin would regulate the liquidity of DDX21 in the PR. We evaluated the fluorescence recovery of EGFP-DDX21 in the PR after Fibrillarin knockdown. However, the recovery rate of EGFP-DDX21 fluorescent intensity after photobleaching was not affected by Fibrillarin depletion (Fig. S9A–C). Therefore, the mobility and localization of DDX21 in the PR are not regulated by Fibrillarin. Our results suggest that there must be other partners to regulate DDX21 liquidity in the PR. Besides, the propensity for LLPS of DDX21 might also be controlled by isoelectric properties of IDRs and post-translational modifications as reported recently [17, 43].

We wondered whether the U3 snoRNP remains in the PR and is regulated by DDX21 or U3 snoRNA. We found that NOP58, the component of the U3 snoRNP complex, formed more aggregates on mitotic chromosomes in U3-depleted cells, while the protein levels of NOP58 remained unchanged (Fig. S10A–D). On the other hand, NOP58 maintained its PR localization after DDX21 knockdown (Fig. S11A–C). These results indicate that the U3 snoRNP complex is present in the PR during mitosis, suggesting that U3 snoRNA might control the PR distribution of other components of pre-ribosomal particles. Our data suggest that U3 snoRNA plays a key role in the PR localization of U3 snoRNP, and regulation between the protein components needs further exploration.

We also noticed that U3 snoRNA forms aggregates on the chromosomes after DDX21 knockdown. Recent studies highlighted the ability of RNA gelation through multivalent RNA-RNA base-pairing [44, 45], implying that the U3 snoRNA aggregates are formed by RNA gelation. The latest research indicates that DEAD-box helicases potentially work as chaperones to limit RNA condensation in stress granule assembly [46]. Besides, U3 snoRNA might also assemble into aggregates together with other PR-localized proteins. We thus presume that DDX21 might function as a chaperone to maintain the proper distribution of U3 snoRNA in the PR, through limiting the excessive intermolecular U3-U3 interactions or U3 snoRNA-protein interactions. However, this needs further exploration.

Our study revealed that the functional domains and motifs of DDX21 are required for its PR localization and mitotic functions. Flag-DDX21△HC disperses in the cytoplasm in mitotic cells. Given that the HC domain contains several highly conserved motifs that bind ssRNA [47], the PR localization of DDX21 might rely on the association with pre-rRNAs, which are the most abundant RNA species in the PR [4]. This speculation was confirmed by the dispersed distribution of DDX21 in the cytoplasm when pre-rRNA transcription was inhibited. On the other hand, Flag-DDX21△GUCT, Flag-DDX21^DAT^ and Flag-DDX21^SAT^ form aggregates on the mitotic chromosomes. Structural analysis of DDX21 revealed that the RRM-containing GUCT domain might facilitate the formation of an RNA-binding unit together with the C-terminal basic tail [30, 48]. Additionally, the GUCT domain mediates protein-protein interactions and makes DDX21 dimer more compact [30, 49]. Hence, the GUCT domain might contribute to the PR distribution by maintaining the compact conformation of DDX21 or mediating the interaction with RNA. It has been recently found that the ATPase activity of DEAD-box ATPases (DDXs) is required for its function in the regulation of the dynamics of RNA-containing phase-separated organelles [50]. Besides, DDXs promote phase separation dependent on ATP-binding activity [50]. Specifically, the ATP-binding motif of DDX21 maintains its closed conformation to control the size and mobility of FC and DFC in the nucleolus [32]. Thus, the ATP-binding ability and ATPase activity might contribute to the PR distribution of DDX21 through controlling its conformational switch and the RNA flux in the PR. We further demonstrated that DDX21 controls mitosis depending on its PR localization, providing novel insights into the roles of PR components in mitotic regulation. Besides, we observed that NOP58 also forms aggregates on chromosomes in cells transfected with Flag-DDX21△HC, Flag-DDX21△GUCT, Flag-DDX21^DAT^ and Flag-DDX21^SAT^ (Fig. S12A, B), indicating that these mutants might also modify the PR distribution of NOP58.

Several functions of PR have been proposed previously: (i) maintaining chromosome structure, particularly in the formation of heterochromatin; (ii) promoting chromosome individualization and clustering [51, 52]; and (iii) acting as a landing pad to carry and distribute client proteins and RNAs following cell division [29]. In the present study, we demonstrate that both U3 snoRNA and DDX21 are required for mitosis, rather than acting as a landing pad. DDX21 controls mitosis through its uniform PR localization, which is regulated by U3 snoRNA. Specifically, U3 snoRNA controls the PR distribution of DDX21 by maintaining its mobility and downsizing the DDX21 aggregates. Besides, our study suggests that the regulation of the phase separation of DDX21 deserves further exploration. In summary, our study provides novel insights for understanding how snoRNA and nucleolar protein function in the PR assembly. Our findings demonstrate that the proper molecular ratio of U3 snoRNA/DDX21 determines mitotic cell fate, and disruption of the molecular balance between U3 snoRNA and DDX21 might provide a potential strategy for tumor therapy through interfering with the PR assembly and causing mitotic catastrophe.

## Materials and methods

### Cell culture and transfection

HeLa, GFP-H2B + RFP-α-Tubulin HeLa and human embryonic kidney (HEK) 293 T cells were cultured in Dulbecco’s modified Eagle’s medium, supplemented with 10% bovine serum. The stable cell line GFP-H2B + RFP-α-Tubulin HeLa was provided by D. W. Gerlich (Institute of Molecular Biotechnology of the Austrian Academy of Sciences, Vienna BioCenter, Vienna, Austria). Cells were incubated in a humidified chamber of 5% CO_2_ at 37 °C. Cells were routinely tested for Mycoplasma contamination. Cells were transfected with plasmid DNA, siRNA duplexes or ASOs by Lipofectamine 2000 (Invitrogen) according to the manufacturer’s instructions.

### Small interfering RNAs and antisense oligonucleotides

All siRNAs and ASOs were purchased from GenePharma (Shanghai, China). For silencing DDX21, siRNA-2 5’-CGGGAAUUAAGUUCAAACGAA-3’, siRNA-a 5’-GAGACUUAAUACUGAGCAAUG-3’ and siRNA-b 5’-GGGUUGUAAUACAGUUUAUAC-3’ were used. For silencing Fibrillarin, siRNA 5’- CGAGAGAUGUGUGUUGAUA-3’ was used. For the knockdown of U3 snoRNA, U3 ASO mUmUmCmGmGTGCTCTACACmGmUmUmCmA was used as described previously [53]. N and mN are deoxynucleotide and 2′-O-methoxyethylribonucleotide, respectively. Phosphodiester backbones are phosphorothioates.

### Plasmid construction

pMS2-GFP and pcDNA3.1-MCS-24*MS2 were purchased from Fenghbio. pcDNA-U3-MS2 was cloned into pcDNA3.1-MCS-24*MS2. U3 snoRNA was cloned into pcDNA3.1 vector. Flag-tagged DDX21 and DDX21 mutants (△GUCT, △HC, DAT, SAT) were cloned into pCI-neo vector. His-tagged DDX21 and DDX21 mutants were cloned into pET-28b (+) vector. All plasmids cloned with PCR products were confirmed by DNA sequencing. Mutant plasmids including Flag-DDX21^DAT^, Flag-DDX21^SAT^, His-DDX21^DAT^ and His-DDX21^SAT^ were obtained by mutagenesis using the Quick-Change Site-Directed Mutagenesis Kit (Stratagene) according to the manufacturer’s protocol. The presence of mutations in the constructed plasmids was confirmed by DNA sequencing.

### Lentiviral production and infection

DDX21 shRNA-a or shRNA-b oligos were cloned into pLKO.1 vector following the protocol (Addgene). pLKO.1-DDX21 shRNA or pLKO.1-ctrl shRNA plasmid was transfected into HEK293T cells with the packaging vectors pMD2.G and psPAX2 to produce lentiviral particles. HeLa cells were infected with lentiviruses delivering DDX21 shRNA or control shRNA to obtain stable DDX21 knockdown and control cell lines. DDX21 was subcloned into pLV-EGFP-N vector. pLV-EGFP-DDX21 was transfected into HEK293T cells with vectors pH1 and pH2 to produce lentiviral particles. HeLa cells were infected with lentiviruses delivering pLV-EGFP-DDX21 to obtain stable DDX21 overexpression cell lines. Stable cell lines were selected by treatment of puromycin (2 μg/mL). The infection efficiency was determined by Western blot.

### Antibodies and reagents

Antibodies used in this study include α-tubulin (immunofluorescent staining: AC007, ABclonal; Western blot: AC012, ABclonal), DDX21 (sc-376953, Santa Cruz Biotechnology), β-actin (AC026, ABclonal), Fibrillarin (A0850, ABclonal), GFP (HT801, TransGen Biotech), Cyclin B1 (sc-245, Santa Cruz Biotechnology), NOP58 (A4749, ABclonal) and Flag (Western blot: HT201, TransGen Biotech; immunofluorescent staining: F3165, Sigma-Aldrich). Thymidine (T1895), actinomycin d (SBR00013) and nocodazole (N219) were purchased from Sigma-Aldrich.

### Cell extraction and Western blotting

Cells were harvested and lysed in a lysis buffer containing 50 mM Tris-Cl pH 7.4, 250 mM NaCl, 0.5% Nonidet P-40, 1 mM PMSF, 1 mM Na_3_VO_4_, 1 mM EDTA, 1 mM NaF and cocktail of protease inhibitors. Following lysate clearance with centrifugation for 30 min at 12,000 rpm and 4 °C, protein quantification was performed with Coomassie Brilliant Blue G250 (Beyotime). Proteins from cellular fractions were separated on SDS-PAGE and transferred onto a 0.45 μm nitrocellulose blotting membrane (Amersham). Membranes were probed with corresponding primary antibodies after blocking with 5% milk in PBS. After extensive washing with PBS/T (0.5% Tween-20 in PBS buffer), the membranes were incubated with IRDye^®^ 800CW or 680RD secondary antibodies (LI-COR). Fluorescence signals were detected using Odyssey^®^ CLx imager. Images were acquired using Image Studio 5.0 (LI-COR).

### Live cell imaging

The mitotic progression of GFP-H2B + RFP-α-Tubulin HeLa cells was monitored in living cells grown in 35 mm dishes with glass bottoms (430165, Corning) under UltraVIEW VoX (PerkinElmer, USA). Images were acquired by using a 63× objective enclosed in a humidified incubation chamber with 5% CO_2_ at 37 °C, and images were captured every 10 min for 20–30 h. The videos were acquired by the Volocity software.

### Cell synchronization

Cells were synchronized by single thymidine block. Briefly, cells were synchronized at the G_1_/S transition after being treated with 2.5 mM thymidine for 24 h. To synchronize the cells in M phase, cells were washed with phosphate-buffered saline (PBS) three times after treatment with 2.5 mM thymidine for 24 h and incubated with 50 ng/mL nocodazole for 12 h. Mitotic cells were collected by shaking off.

### Immunofluorescence and RNA fluorescence in situ hybridization (FISH)

For immunofluorescence assays, HeLa cells grown on glass coverslips were fixed with ice-cold 4% paraformaldehyde (PFA) in PBS and were permeabilized in 0.5% Triton X-100. Cells were then blocked in 5% goat serum and 0.1% Triton X-100 in PBS and incubated with appropriate primary antibodies at 4 °C overnight. After washing with PBS, cells were incubated with secondary antibodies conjugated with DyLight 488 or DyLight 594 (1:100) (Earthox). The nucleus and chromosomes were stained with DAPI (Beyotime) and the coverslips were mounted. Images were acquired using confocal microscopy (TCS-SP8 DIVE, Leica). Cellular counting and intensity evaluation were performed using ImageJ.

For U3 snoRNA FISH, the probe was designed as previously described [54] with slight modifications and labeled with Cy3 on the 5’ and 3’ ends (U3 probe: CGTTCTCTCCCTCTCACTCCCCAATACGGAGAGAAGAACGATCATCAATGGCTGA). For FISH detecting the localization of pre-rRNAs, the probe was designed according to the RT-qPCR primers as previously described [3], and labeled with Cy3 on the 5’ and 3’ ends (pre-rRNA probe: CTCCAGGAGCACCGCAAGGGC). The probes were synthesized in Tsingke Biotechnology (China). Cells were fixed with 4% PFA, followed by permeabilization with 0.5% Triton X-100. Cells were incubated with 200 ng/mL Cy3-labeled U3 or 5’ ETS probe in hybridization buffer (2×SSC, 50% deionized formamide, 10% dextran sulfate, 1×Denhardt’s solution, 12.5 μg/mL ssDNA) at 37 °C overnight. After hybridization, cells were rinsed twice with 50% formamide in 2×SSC at 37 °C, followed by two washes with 2×SSC at 37 °C for 10 min. Cells were then washed with PBS and stained with DAPI. Slides were mounted, and photos were taken under confocal microscopy (TCS-SP8 DIVE, Leica).

### MS2 pull-down assay and mass spectrometry

HeLa cells were co-transfected with pcDNA3.1-MS2 vector or pcDNA3.1-U3-MS2, and pMS2-GFP using Lipofectamine 2000. Twenty-four hours after transfection, cells were synchronized by 50 ng/mL nocodazole for 12 h. Cells were harvested and lysed in Buffer A (25 mM Tris-Cl pH 7.5, 150 mM KCl, 1 mM DTT, 2 mM EDTA, 0.5 mM PMSF, 0.2% Nonidet P-40 and fresh protease inhibitor cocktail). Anti-GFP antibody was coupled with a 50% suspension of protein A-Sepharose beads (Cytiva) in IPP500 (500 mM NaCl, 10 mM Tris-Cl pH8.0, 0.2% Nonidet P-40) for 2 h at 4 °C. After washing, the coupled beads were incubated with 5 mg cell lysates for 4 h at 4 °C. After washing with Buffer B (10 mM Tris-Cl pH 8.0, 150 mM KCl, 5 mM MgCl_2_, 0.1% Nonidet P-40) five times, the precipitants were subjected to SDS-PAGE and visualized by silver staining. The bands were cut from SDS-PAGE gel, fully trypsinized, and analyzed by Q-Extractive liquid chromatography-tandem mass spectrometry (LC-MS/MS).

### Bioinformatics

The gene sets enrichment analysis was performed on the Metascape website (http://metascape.org). U3 snoRNA-binding proteins were predicted on the RNAct website (http://rnact.crg.eu).

### RNA immunoprecipitation (RIP)

The RIP assay was performed as previously described [55]. Briefly, cells were extracted in lysis buffer containing 25 mM Tris-HCl pH7.5, 150 mM KCl, 2 mM EDTA, 0.5% NP-40, 1 mM NaF, 1 mM DTT, 100 U/mL RNasin and EDTA-free protease inhibitor. The protein A-Sepharose beads coated with anti-DDX21 antibodies were incubated with cell lysates overnight at 4 °C. After being washed five times, the DDX21-immunoprecipitated complexes were incubated with proteinase K digestion buffer. RNA was extracted by TRIzol-chloroform RNA extraction methods. The relative expression of RNA was determined by RT-PCR and normalized to the input. Normal mouse IgG was used as a negative control.

### Quantitative real-time PCR

Total RNA was extracted from cells using TRIzol reagent (Invitrogen). cDNA was synthesized from total RNA using Hifair^®^ III 1st Strand cDNA Synthesis SuperMix (YEASEN). The quantitative polymerase chain reaction (qPCR) analysis was performed using the Hieff UNICON^®^ Universal Blue qPCR SYBR Green Master Mix (YEASEN) and ABI 7500/7500 Fast Real-time PCR System (Applied Biosystems). The human β-actin mRNA was amplified as an internal control. All real-time PCR data were analyzed by comparative C_t_ method and normalized to β-actin.

### Fluorescence recovery after photobleaching (FRAP) assay

The FRAP assays were performed as previously described [32]. HeLa cells stably expressing EGFP-DDX21 were cultured in 35 mm glass-bottom dishes (Cellvis) 24 h before imaging. FRAP assay was performed with the Leica STED laser confocal microscopy equipped with a 63 × oil immersion objective. GFP signals in regions of interest (ROI) were bleached using an OPSL 488 nm laser. The laser power of bleaching was optimized to obtain an efficient bleaching effect. Fluorescence recovery was monitored for 90 s at intervals of 2 s. The fluorescence intensity including pre-bleaching and each time point after bleaching was recorded by microscope. The FRAP data was recorded and quantified by Leica LAS X software. The fitting curves were generated using Prism 9.0.

### Protein purification

The expression plasmid for His-tagged DDX21 was transformed into *Escherichia coli* strain BL21 (DE3, TransGen), and the protein expression was induced by 0.1 mM of isopropy-β-D-thiogalactoside (IPTG) for 4 h at 37 °C. The *E. coli* cells were resuspended in lysis buffer (20 mM Tris-Cl pH 7.5, 500 mM KCl, 10% glycerol, 0.1% Triton X-100, 5 mM imidazole, fresh protease inhibitor cocktail), followed by centrifugation and ultrasonication. His-DDX21 was purified by Ni-NTA Agarose (QIAGEN) and was eluted with 250 mM imidazole. Purified proteins were further treated with 0.1 mg/ml RNase A (TIANGEN) to remove RNA. The proteins were then concentrated by Amicon Ultra centrifugal filters (Millipore). Proteins were resolved by Coomassie Blue staining after separating on SDS-PAGE.

### Flow cytometric analysis

For DNA content analysis, cells were trypsinized, washed with cold PBS and fixed in 70% ice-cold ethanol at 4 °C overnight. Cells were rehydrated in PBS on the second day. Following RNase digestion, cells were stained with propidium iodide. Flow cytometry analysis was performed using red emission at 630 nm. Data from 10000 cells were collected and analyzed by using CellQuest software (Becton Dickinson). For apoptosis analysis, cells were double-stained with Annexin V-FITC and propidium iodide (KeyGen) and subjected to flow cytometric analysis. Quantification of apoptotic cells are summarized from three independent experiments.

### In vitro transcription of U3 snoRNA

DNA template was obtained by single restriction digest of pcDNA3.1-U3 plasmid. To synthesize Cy5-labelled U3 snoRNA, U3 snoRNA was in vitro transcribed using 500 ng linearized pcDNA3.1-U3 plasmid and Cy5-UTP (APExBIO) according to the instruction of the manufacturer (Lucigen). RNase-free DNase was added to remove the DNA template, and Cy5-U3 snoRNA was purified by LiCl precipitation.

### In vitro droplet assay

Purified His-DDX21 was added to the droplet assay buffer containing 30 mM Tris HCl pH 7.4, 100 mM NaCl, 2% Glycerol and 1 mM DTT as previously described [41]. The reaction was performed in 100 μl volumes. Protein was mixed with Cy5-U3 snoRNA first in high salt storage buffer for 1 h at room temperature and was diluted in droplet assay buffer. After incubation at room temperature for 1 h without any shaking or rotating, the mixtures were individually transferred into a 384-well plate (Cellvis P384-1.5H-N) for observation under confocal microscopy (TCS-SP8, Leica) at 63× magnification. The size of each droplet or amorphous condensate was measured by Leica LAS X software.

### Quantification of U3 snoRNA copy number

The copy number of in vitro transcribed Cy5-U3 snoRNA in 100 μL droplet assay buffer was calculated by the DNA/RNA Copy Number Calculator website (http://endmemo.com/bio/dnacopynum.php).

### Quantification of DDX21 copy number

His-DDX21 concentration was analyzed by Coomassie Blue staining. The copy number of His-DDX21 in 100 μL droplet assay buffer was calculated following the formula [32]: n_DDX21_ = m_DDX21_×N_A_/M_DDX21_. m_DDX21_: DDX21 protein mass. N_A_: Avogadro constant. M_DDX21_: DDX21 molecular weight.

### Statistical analysis

All data analyses were performed using Prism 9.0 (GraphPad Software, USA). Two-tailed unpaired Students’ *t*-tests were used to compare the differences between two groups. One-way analysis of variance (ANOVA) was used to analyze differences among more than two groups. All data met the assumptions of the tests and were presented as mean ± SD. *P* < 0.05 was considered to indicate statistical significance. *P < 0.05, **P < 0.01, ***P < 0.001 and ****P < 0.0001 for all analyses.

### Supplementary information

Supplementary materials

Supplementary video 1

Supplementary video 2

Supplementary video 3

Supplementary video 4

Supplementary video 5

Table S1

Table S2

Table S3

Original Western blots

## Data Availability

All data generated and analyzed in this study are included in the paper and the supplementary materials.
