# Supplementary material for: U3 snoRNA inter-regulates with DDX21 in the perichromosomal region to control mitosis
Source: Cell Death Dis. 2024 May 17;15(5):342. doi: 10.1038/s41419-024-06725-3 (PMC11101645; doi:10.1038/s41419-024-06725-3)
Supplement: Supplementary file 1 — Supplementary materials [file 41419_2024_6725_MOESM1_ESM.docx]

**Supplementary materials**


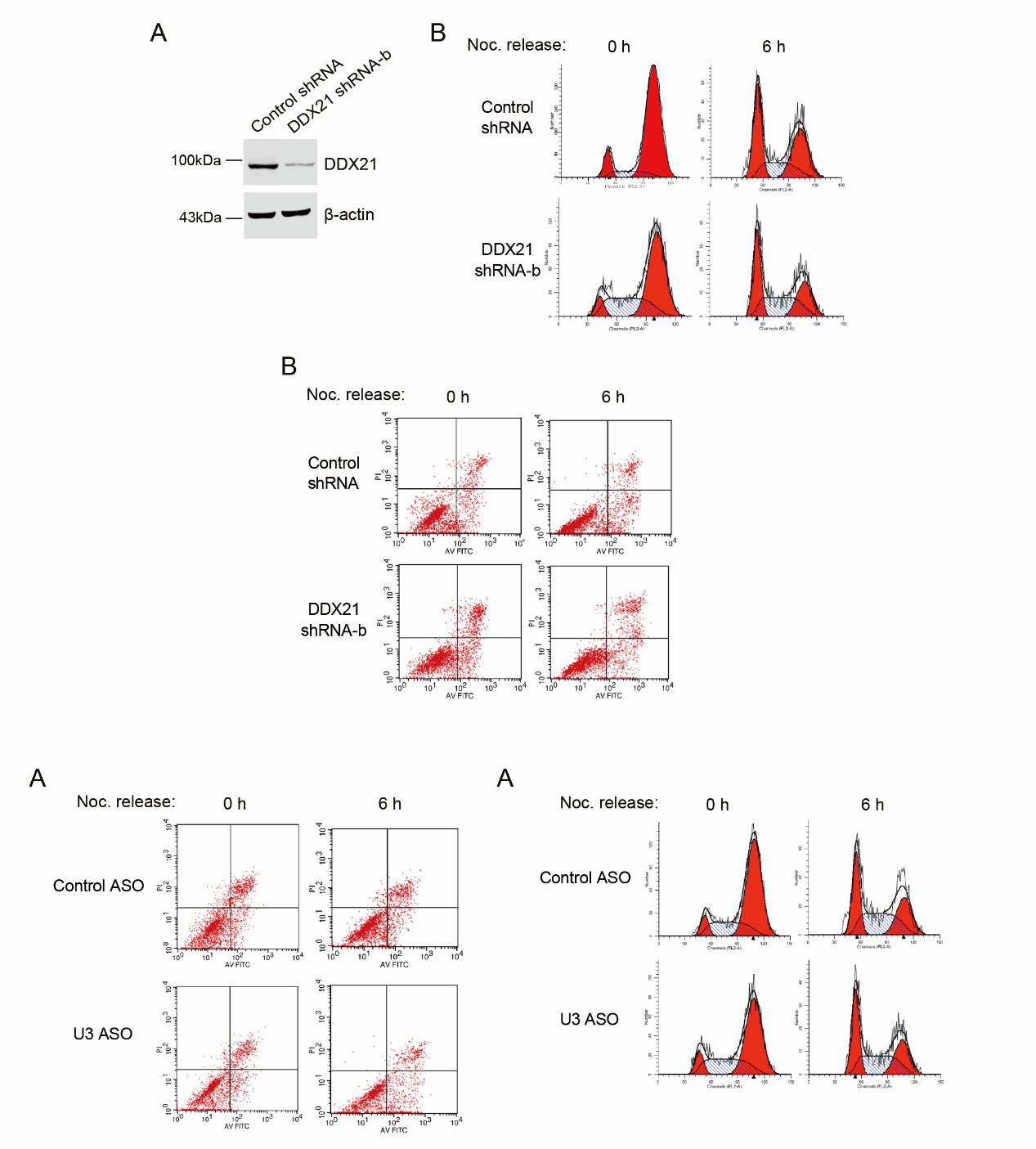


**Fig. S1 Cell cycle determination of U3 snoRNA-depleted cells after released from Thymidine-Nocodazole arrest. A** HeLa cells were transfected with indicated ASOs. Cells were then synchronized at M phase by Thymidine-Nocodazole treatment and collected by mitotic shake-off. Mitotic cells were collected immediately for flow cytometry analysis or released into fresh medium for 6 hours. Cells were fixed and stained with PI. Cell cycle was determined by flow cytometry.


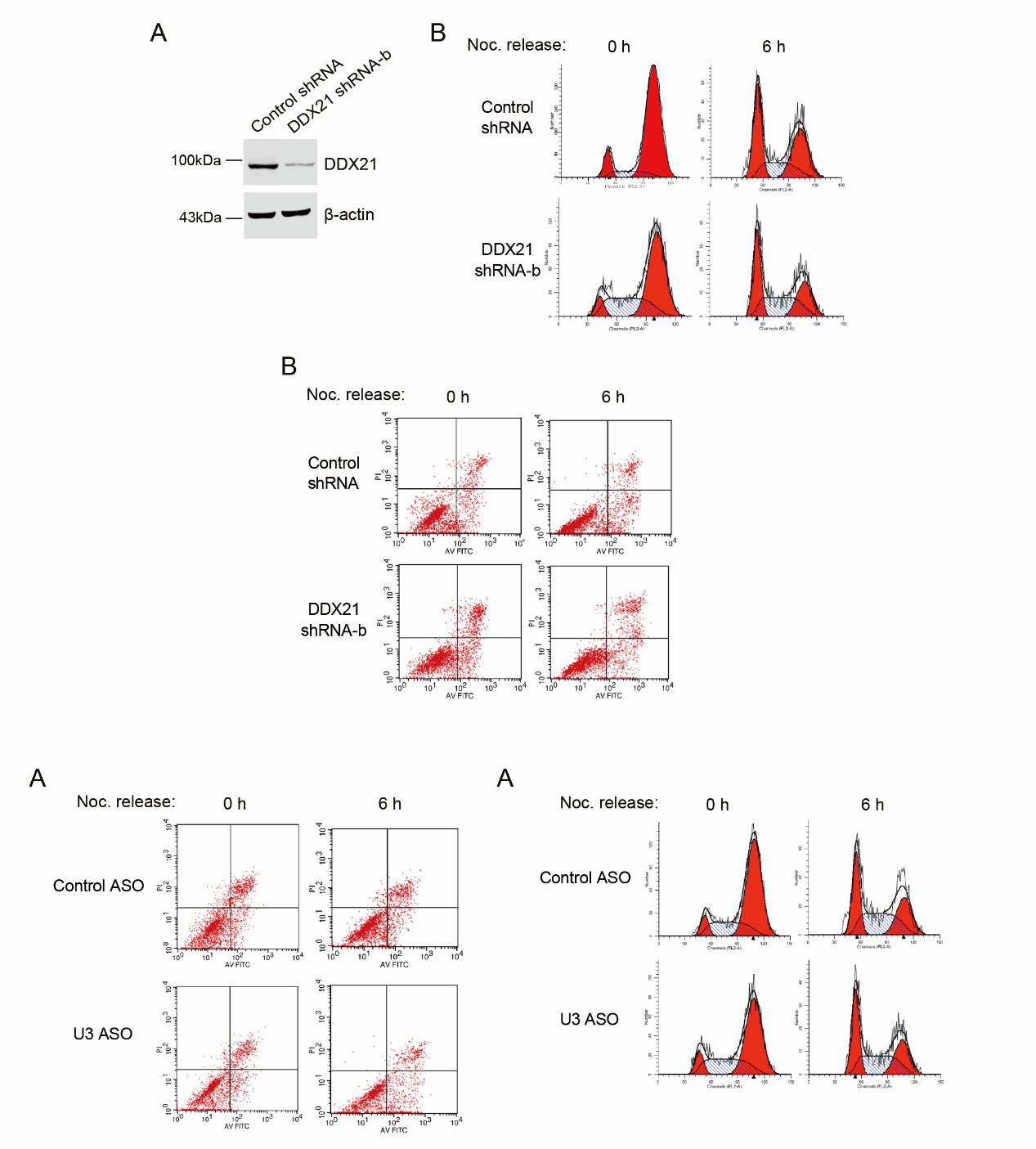


**Fig. S2 Cell cycle determination of DDX21 knockdown cells after released from Thymidine-Nocodazole arrest. A** Whole cell lysate extracted from the DDX21 shRNA cells was subjected to Western blot and probed with indicated antibodies. **B** Control shRNA HeLa cells and DDX21 shRNA-b HeLa cells were synchronized at M phase by Thymidine-Nocodazole treatment and collected by mitotic shake-off. Cells were collected immediately or released into fresh medium for 6 hours. Cell cycle was analyzed by flow cytometry.


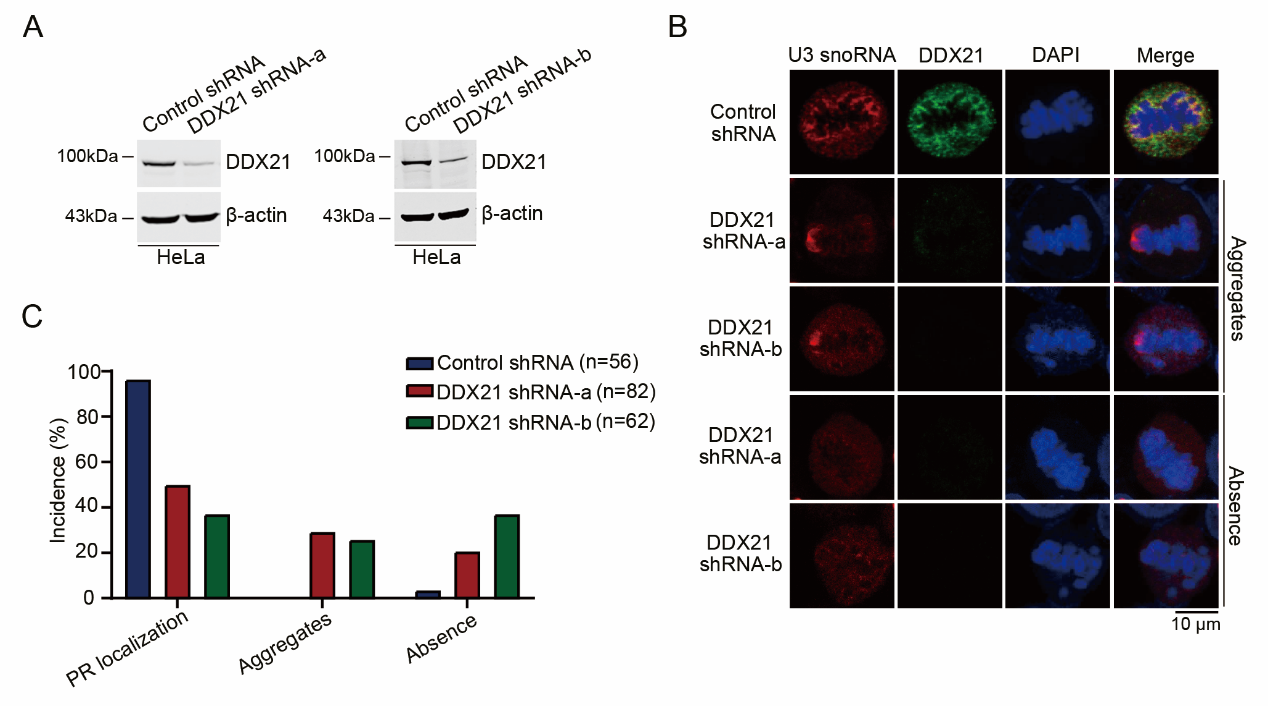


**Fig. S3 DDX21 regulates the PR localization of U3 snoRNA.** **A** Whole cell lysate extracted from the DDX21 shRNA cells was subjected to Western blot and probed with the indicated antibodies. **B** The localization of U3 snoRNA was determined by FISH, and that of DDX21 was determined by immunofluorescent staining. **C** Shown are the U3 snoRNA localization patterns summarized from repeated experiments. Scale bar, 10 μm.


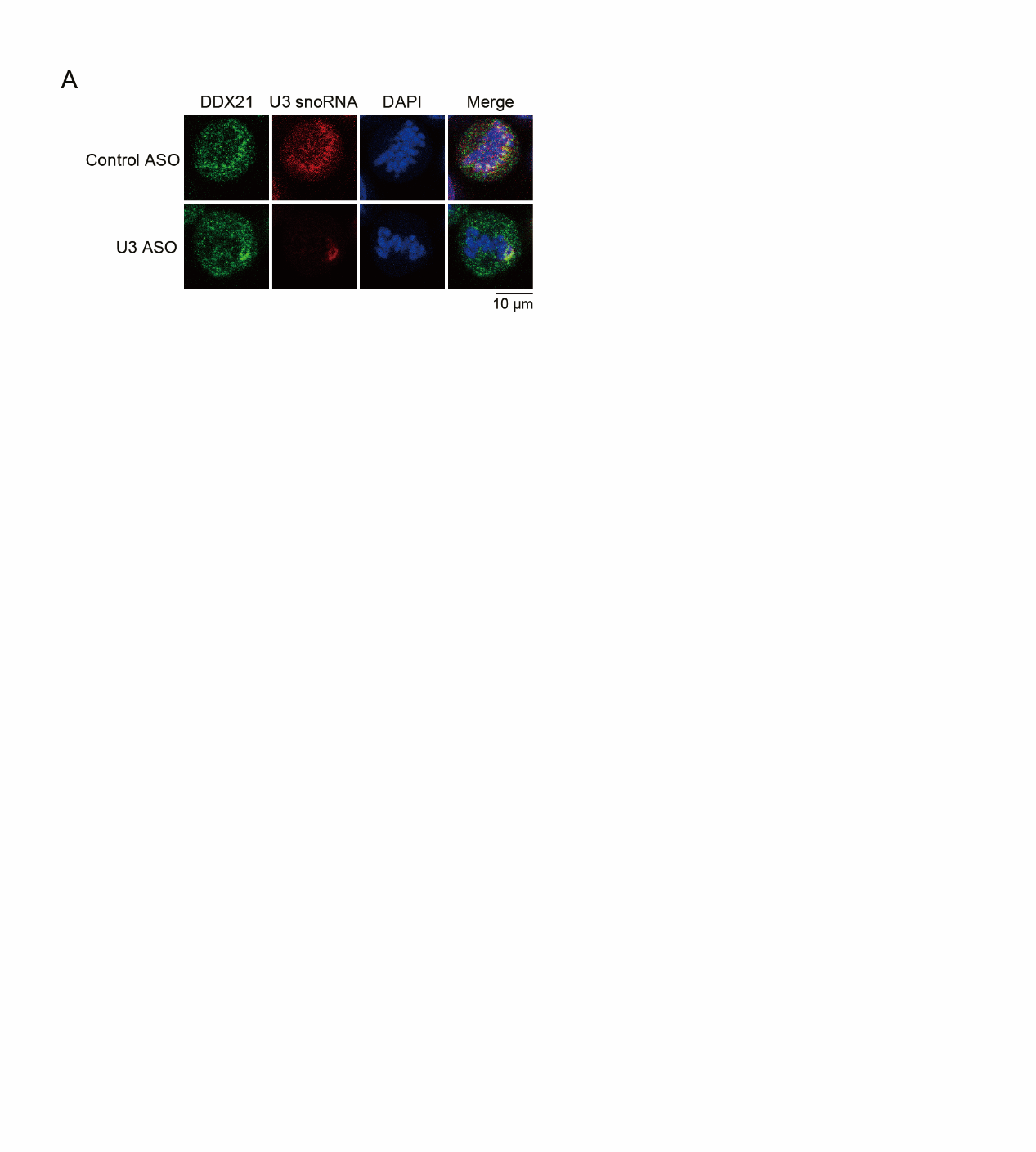


**Fig. S4 DDX21 forms aggregates on mitotic chromosomes after U3 snoRNA depletion. A** HeLa cells were transfected with indicated ASOs. Forty-eight hours later, cells were fixed. FISH was performed for U3 snoRNA staining and immunofluorescent staining was performed to determine the localization of DDX21. Chromosomes were stained with DAPI. Scale bar, 10 μm.


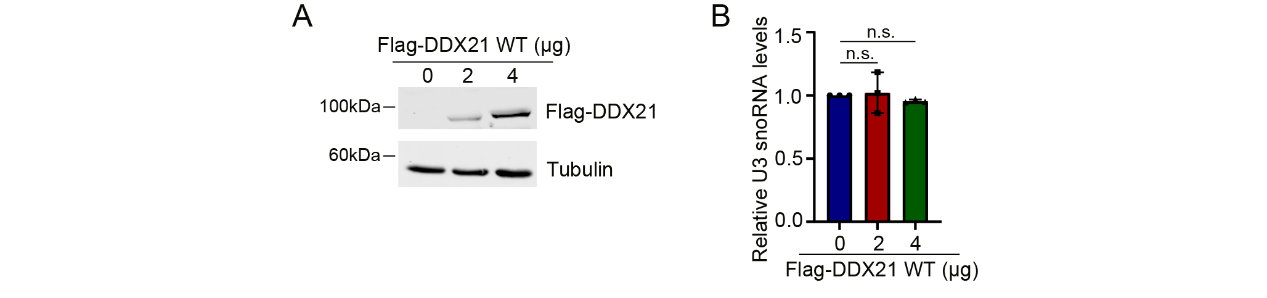


**Fig. S5 U3 snoRNA level is not affected by ectopic Flag-DDX21.** **A** HeLa cells were transfected with increasing amounts of Flag-DDX21^WT^ plasmid. Forty-eight hours later, proteins from cell lysates were immunoblotted with the indicated antibodies. **B** Total RNAs were extracted from HeLa cells described in (**A**). U3 snoRNA levels were evaluated by RT-qPCR. *P*-values were calculated by one-way ANOVA. n.s. denotes no significance.


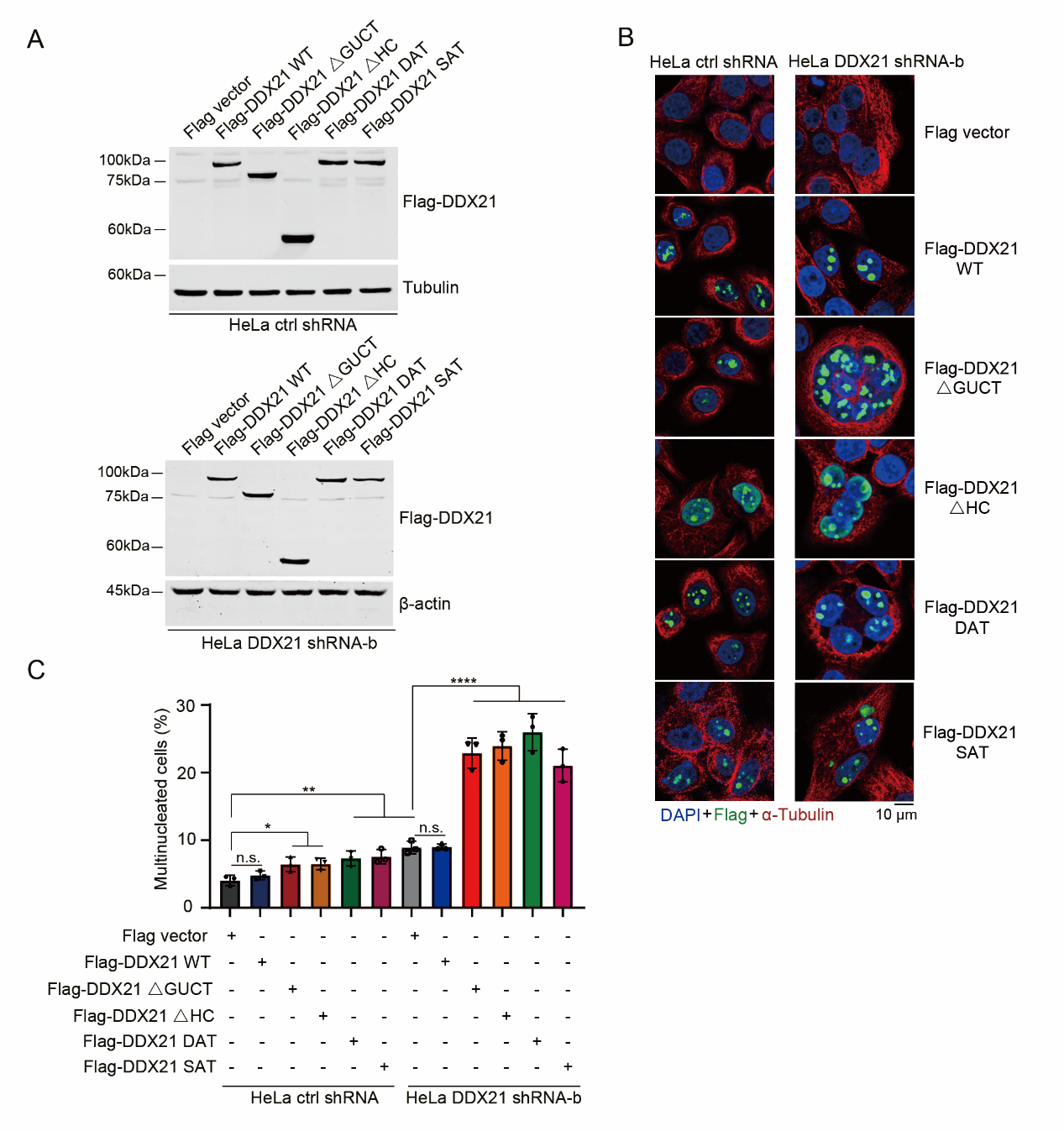


**Fig. S6 Ectopic Flag-DDX21 mutants aggravates the DDX21 depletion-induced multinucleation. A** HeLa DDX21 shRNA-b and control shRNA cells were transfected with indicated plasmids. Proteins from cell lysates were subjected to immunoblot probed with anti-Flag antibody. **B** Cells described in (**A**) were fixed and co-immunostained with anti-Tubulin and anti-Flag antibodies. Nuclei were stained with DAPI. Scale bar, 10 μm. **C** The percentages of multinucleated cells (n > 300) in the above-described cells are calculated. Data was summarized from three independent experiments. *P*-value was calculated using one-way ANOVA. **P* < 0.05. ***P* < 0.01. *****P* < 0.0001. n.s. denotes no significance.

**
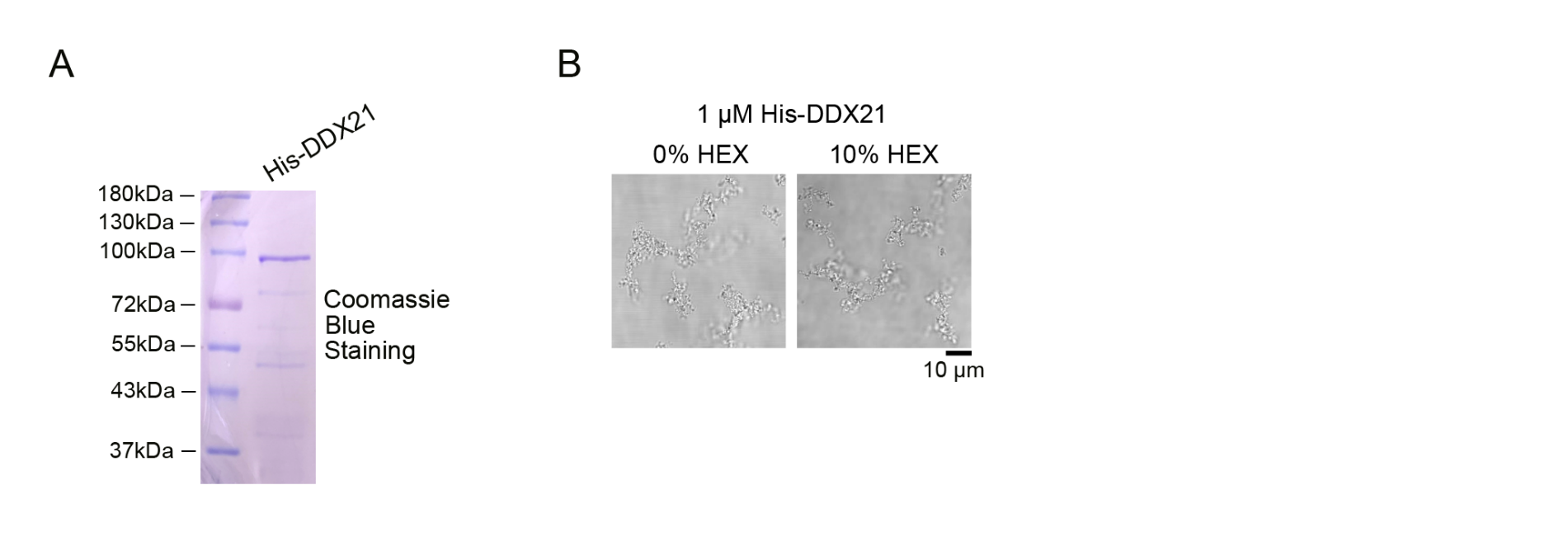
**

**Fig. S7 Purified His-DDX21 protein was resistant to HEX treatment.** **A** Purified His-DDX21 protein was resolved by SDS-PAGE and stained with Coomassie Bright Blue. **B** His-DDX21 (1 μM) was incubated with droplet assay buffer with or without 10% HEX and droplet formation assay was performed. Representative images were shown. Scale bar, 10 μm.


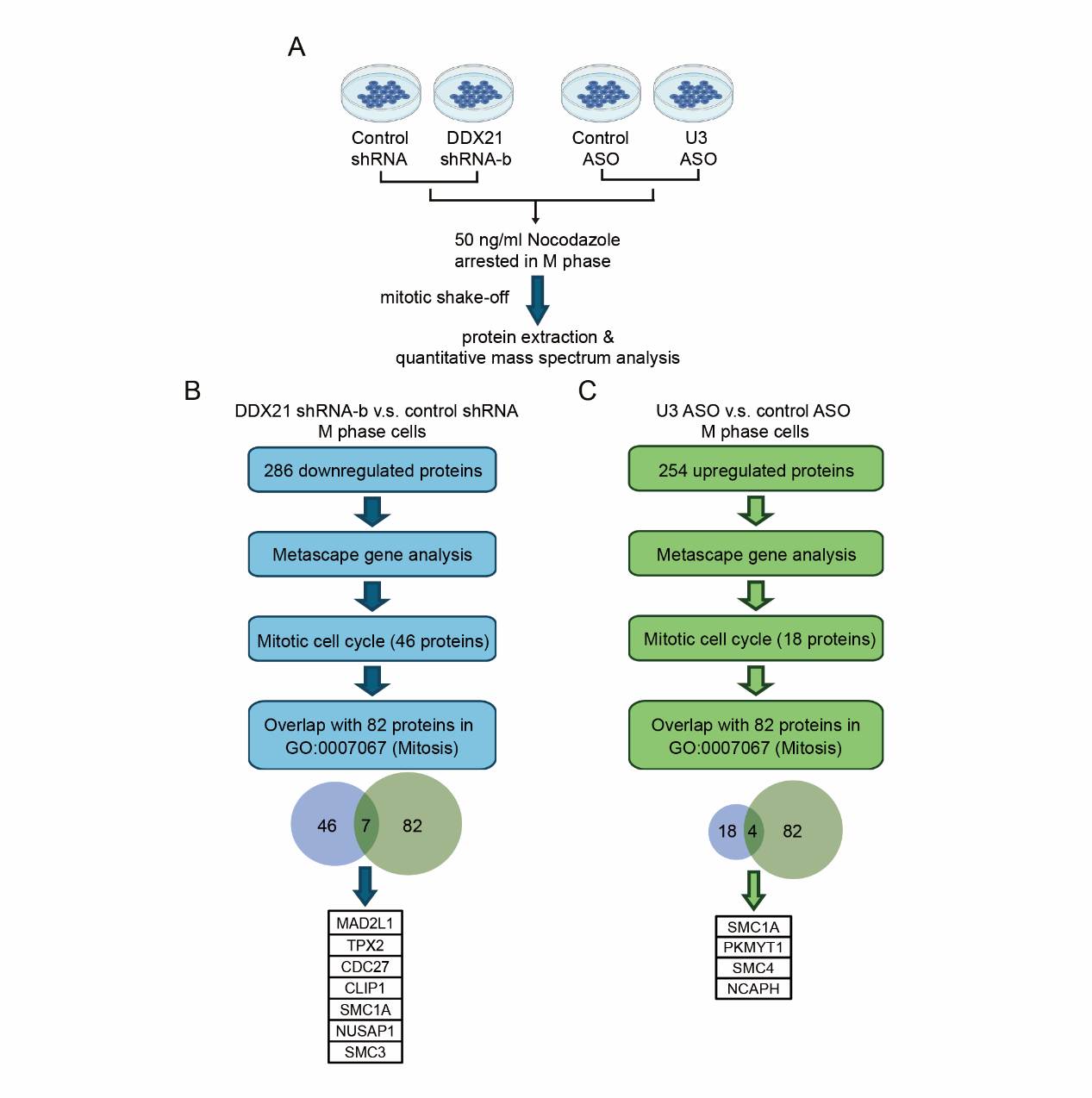


**Fig. S8 Proteomic analysis of mitosis-related genes after DDX21 knockdown or U3 snoRNA depletion. A** Flowchart of collecting mitotic HeLa cells for mass spectrum analysis. **B** and **C** Flowchart of screening mitosis-related genes with significant differences in expression levels between two groups of cells.


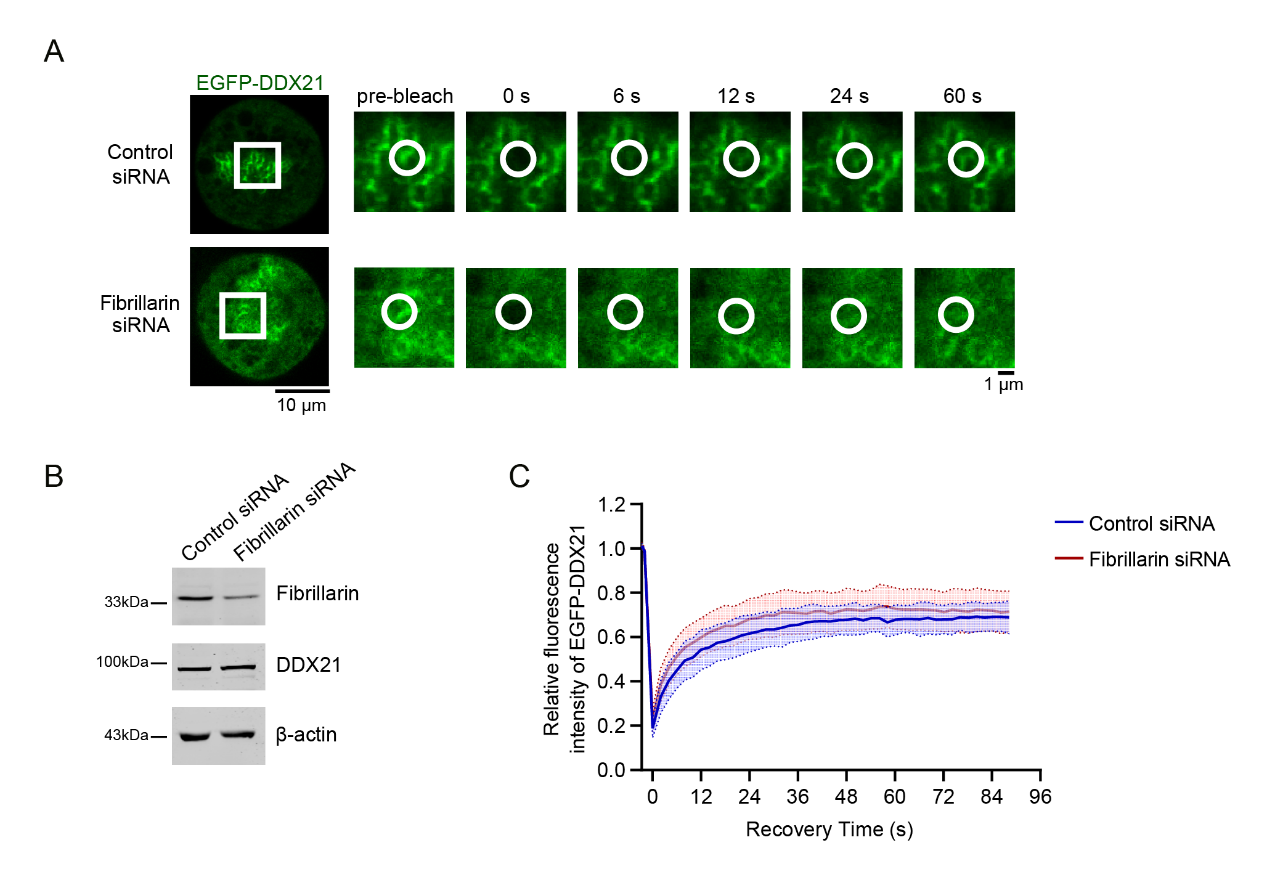


**Fig. S9 The mobility of DDX21 in the PR is independent of Fibrillarin.** **A** HeLa cells stably expressing EGFP-DDX21 were transfected with Fibrillarin siRNA or control siRNA. Seventy-two hours later, FRAP analyses were performed. Left panel: representative snapshots of EGFP-DDX21 in mitotic cells; scale bar, 10 μm. Right panel: magnified views of the region in the white box at different time points (s); the white circles label the regions that were bleached; scale bar, 1 μm. **B** Proteins from cell lysates of cells described in (**A**) were subjected to immunoblot using indicated antibodies. **C** Quantification of the relative fluorescence intensity of EGFP-DDX21 over time in FRAP experiments. Data are presented as mean ± SD, n > 20.


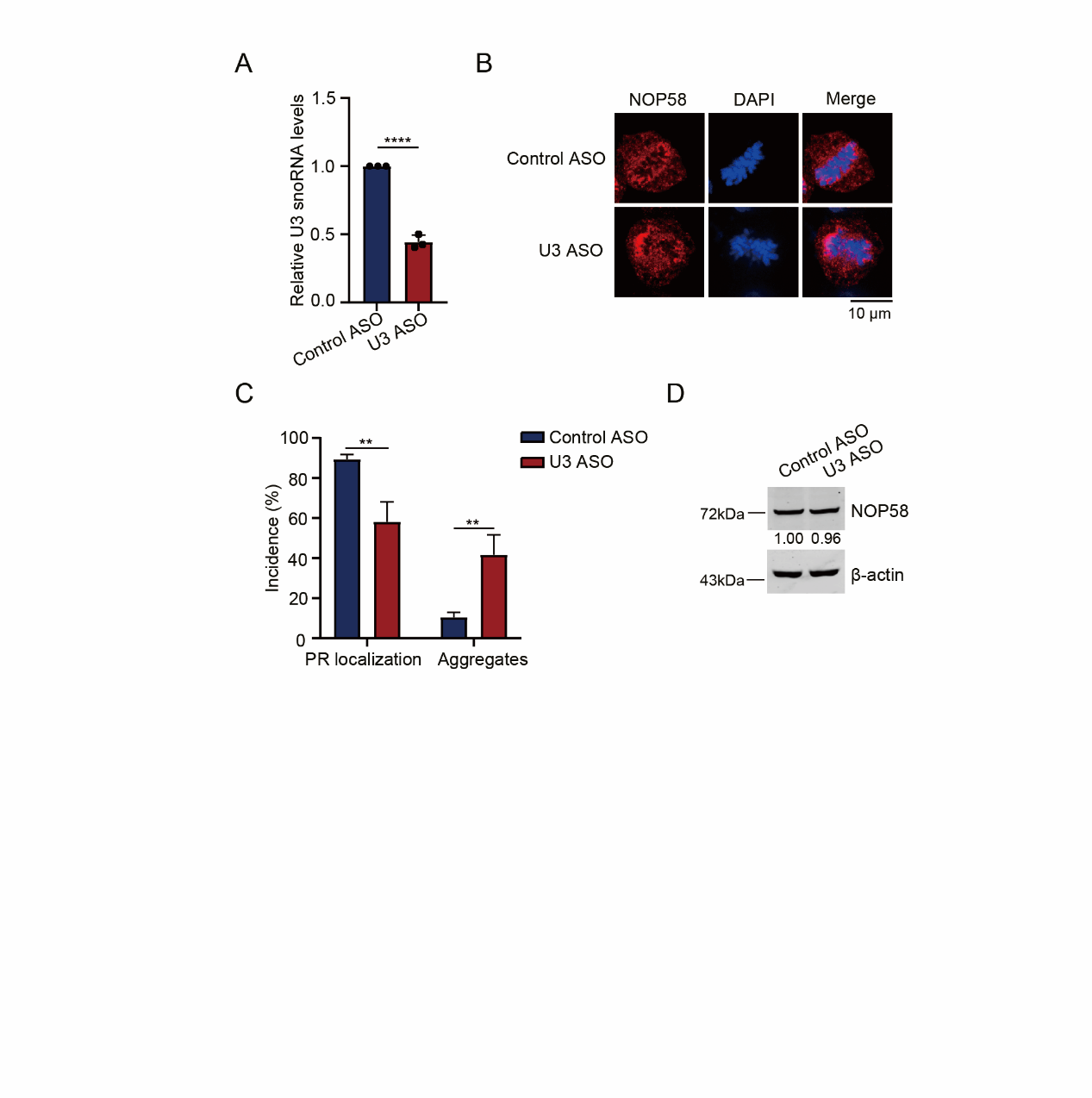


**Fig. S10 NOP58 forms aggregates on mitotic chromosomes after U3 snoRNA depletion. A** HeLa cells were transfected with indicated ASOs. Forty-eight hours later, total RNAs were extracted, and U3 snoRNA levels were evaluated by RT-qPCR. *****P* < 0.0001. **B** Cells described in (**A**) were fixed and immunostained with anti-NOP58 antibody. Chromosomes were stained with DAPI. Scale bar, 10 μm. **C** The incidences of each pattern of NOP58 localization in metaphase cells were summarized. Data was summarized from three independent experiments. n > 40. ***P* < 0.01. **D** Whole cell lysate extracted from cells described in (**A**) was subjected to the Western blot and probed with indicated antibodies.


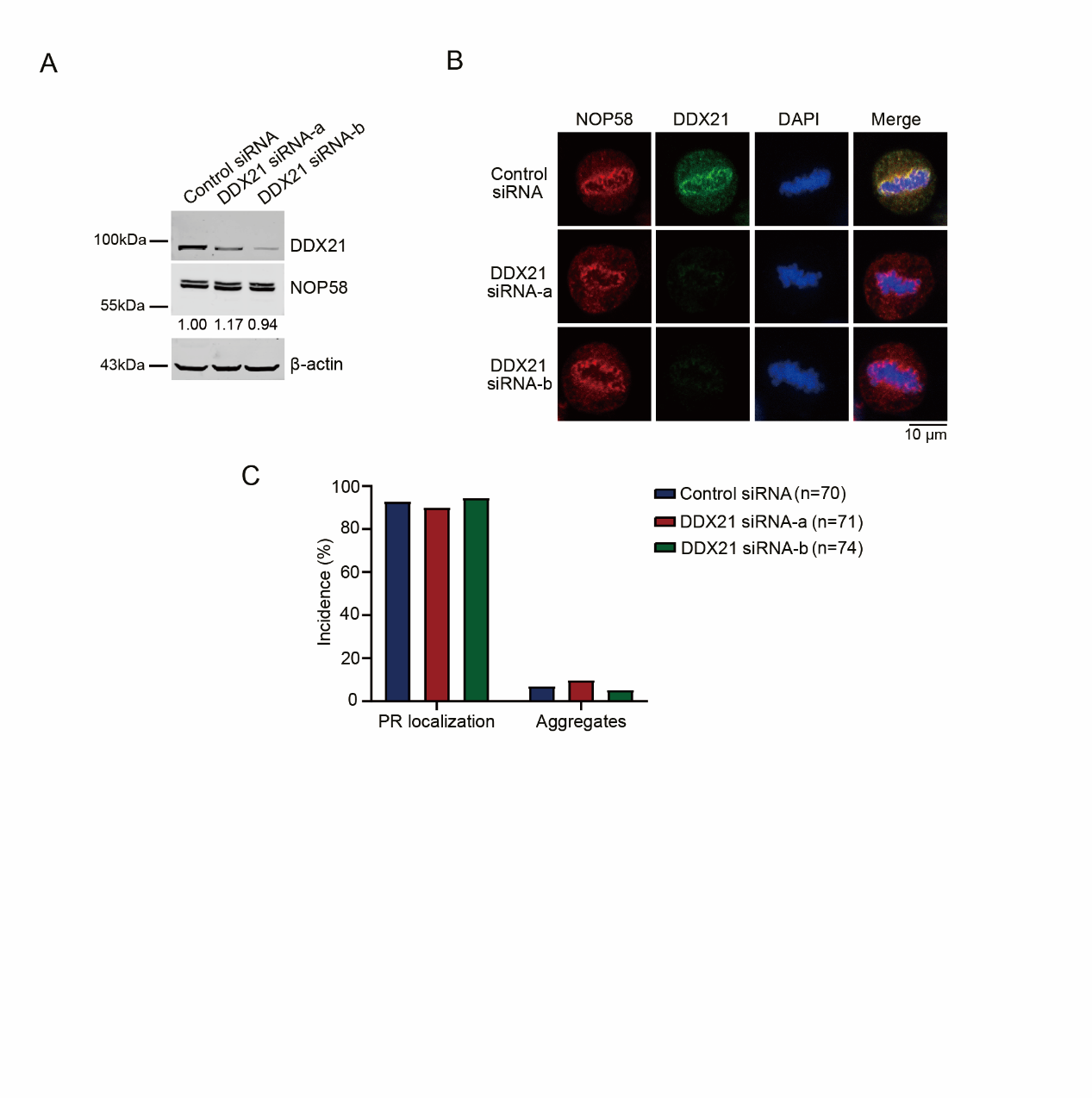


**Fig. S11 The levels and PR localization of NOP58 is independent of DDX21. A** HeLa cells were transfected with indicated siRNAs. Seventy-two hours later, proteins from cell lysates were immunoblotted with indicated antibodies. **B** Cells described in (**A**) were fixed and immunostained with indicated antibodies. Chromosomes were stained by DAPI. Scale bar, 10 μm. **C** The incidences of each pattern of NOP58 localization in metaphase cells were summarized.


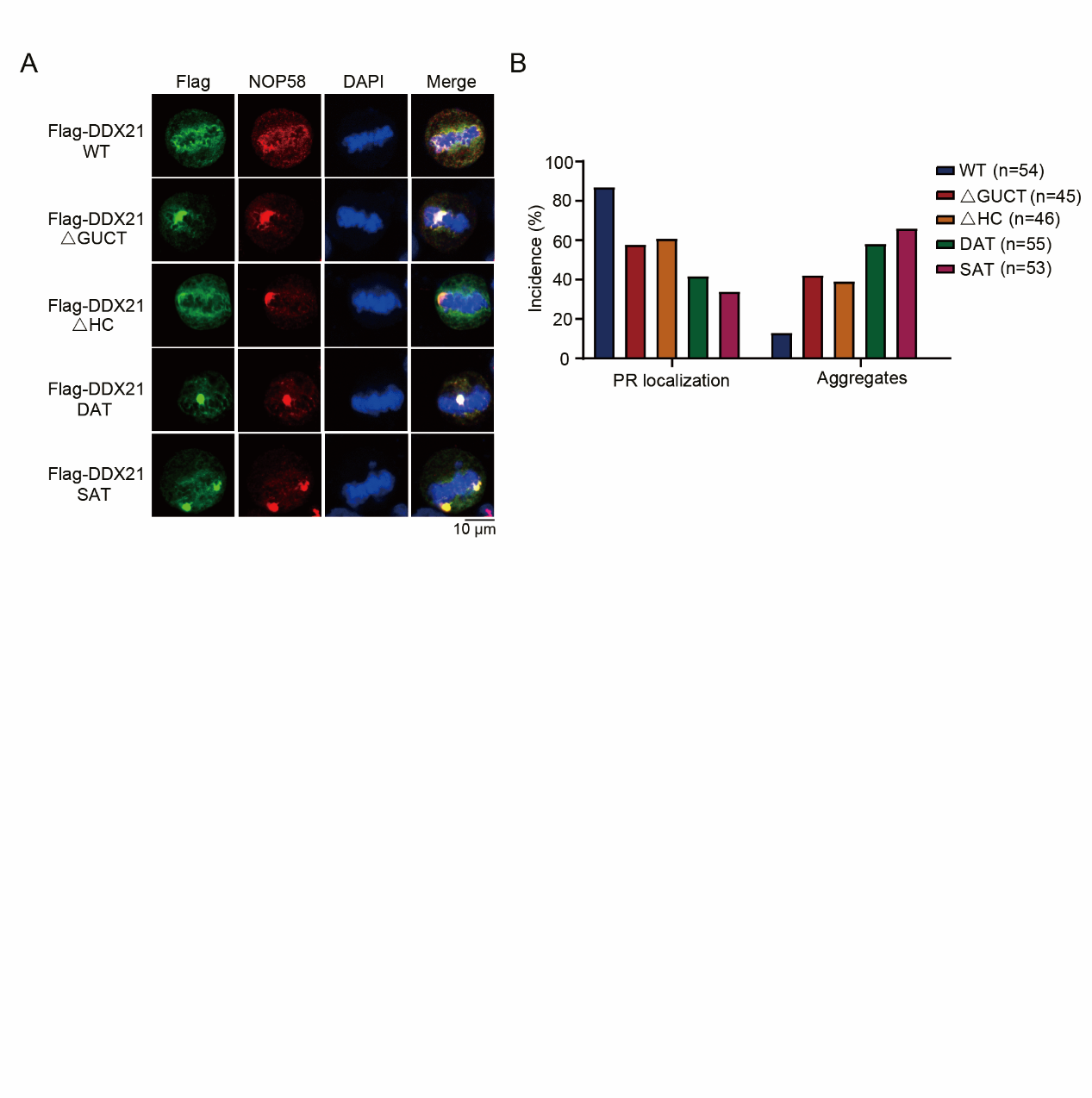


**Fig. S12 Ectopic Flag-DDX21 mutants led to aggregates of NOP58 on mitotic chromosomes.** **A** HeLa cells were transfected with indicated plasmids. Forty-eight hours later, cells were fixed, and immunofluorescent staining was performed using anti-Flag and anti-NOP58 antibodies. Chromosomes were stained with DAPI. Scale bar, 10 μm. **B** The incidences of each pattern of NOP58 localization in metaphase cells were summarized.

**Table Legends**

Table S1. Gene sets enrichment and U3-binding proteins from MS analysis and RNAct prediction.

Table S2. Quantitative mass spectrum analysis data of mitotic control shRNA and DDX21 shRNA-b cells.

Table S3. Quantitative mass spectrum analysis data of mitotic control ASO and U3 ASO cells.

**Video Legends**

Video 1. Mitotic progression of GFP-H2B+RFP-α-tubulin HeLa cells transfected with control ASO.

Video 2. Mitotic process of GFP-H2B+RFP-α-tubulin HeLa transfected with U3 ASO.

Video 3. The video of mitosis of GFP-H2B+RFP-α-tubulin HeLa transfected with control siRNA.

Video 4. The video of mitosis of GFP-H2B+RFP-α-tubulin HeLa transfected with DDX21 siRNA-2.

Video 5. Mitotic progression of GFP-H2B+RFP-α-tubulin HeLa transfected with DDX21 siRNA-a.
