# Supplementary material for: U3 snoRNA inter-regulates with DDX21 in the perichromosomal region to control mitosis
Source: Cell Death Dis. 2024 May 17;15(5):342. doi: 10.1038/s41419-024-06725-3 (PMC11101645; doi:10.1038/s41419-024-06725-3)

Source Data

Figure 2.E


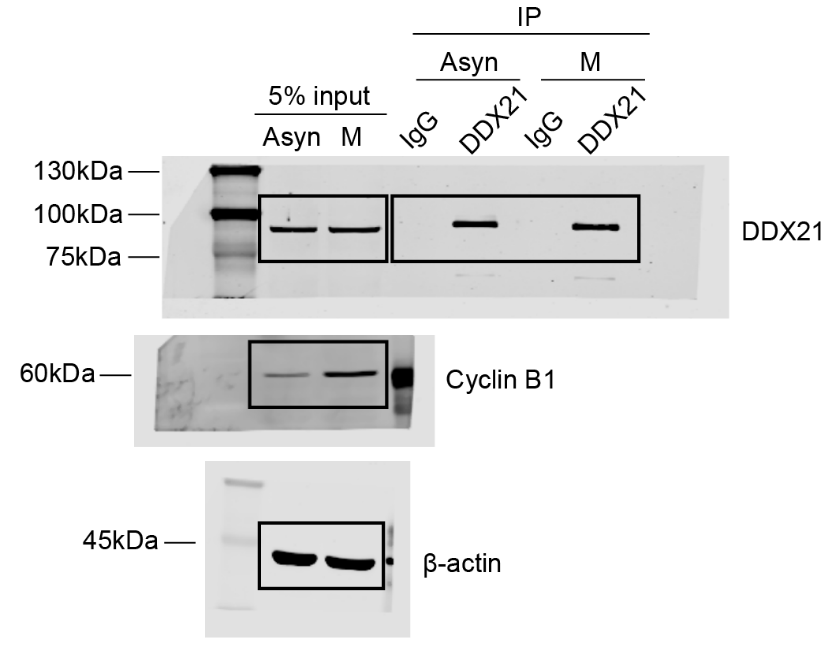


Figure 3.A


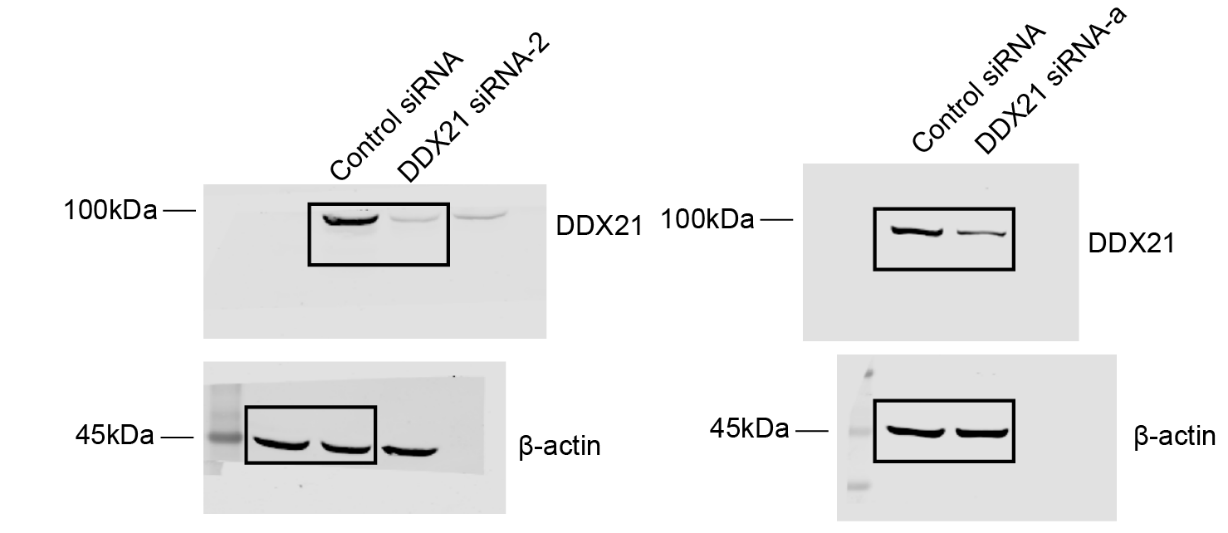


Figure 3.E


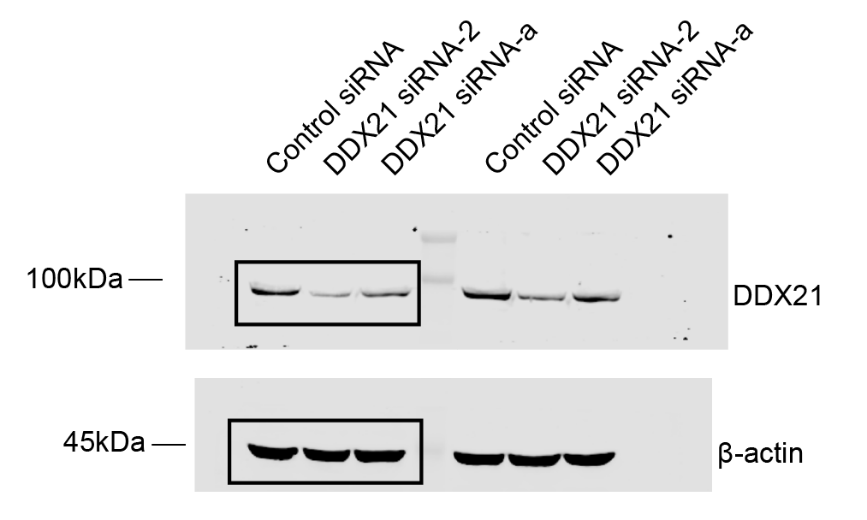


Figure 4.A


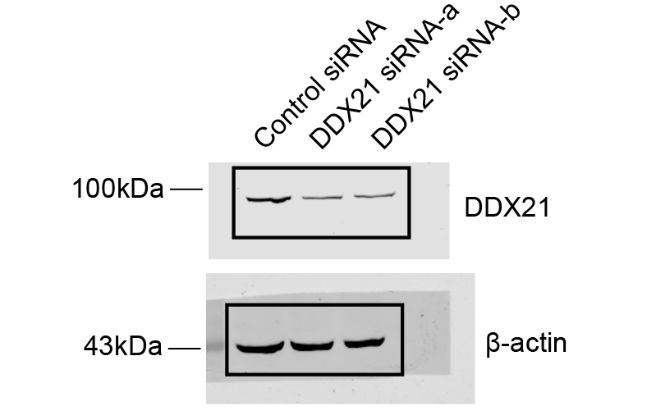


Figure 4.H


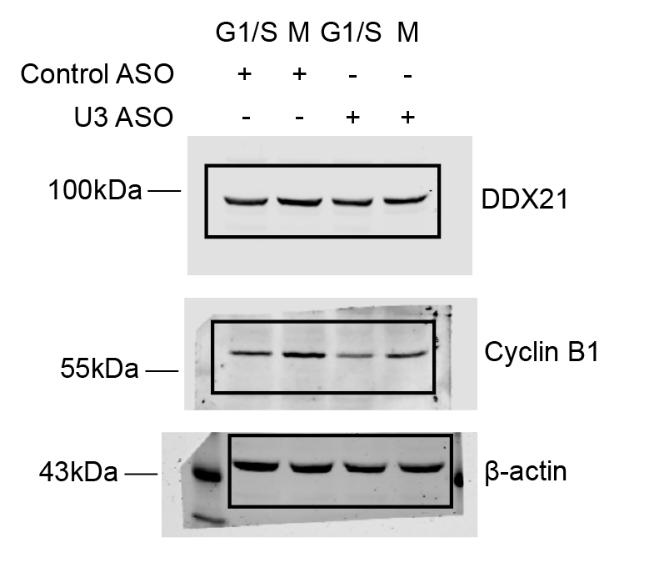


Figure 5.A


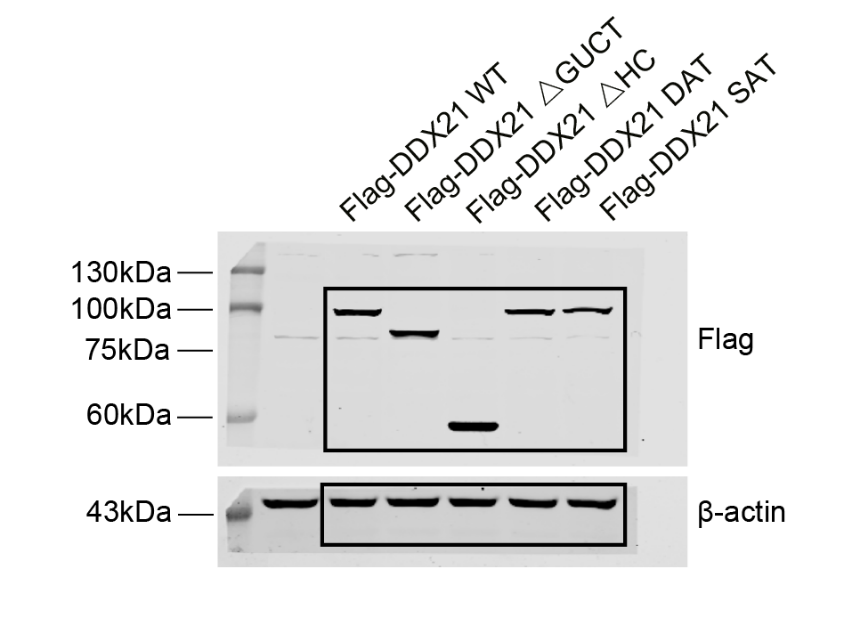


Figure 8.D


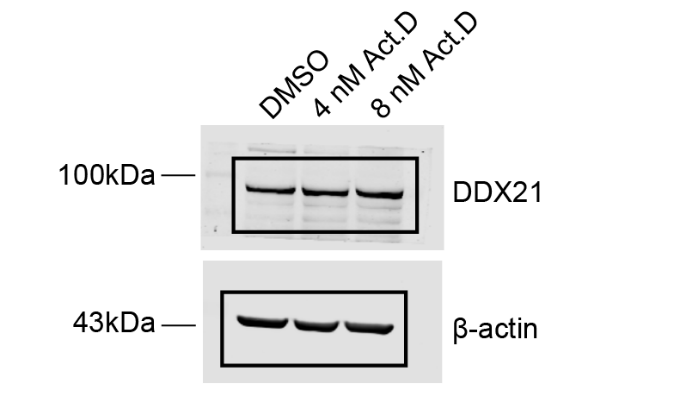


Supplementary Figure S2.A


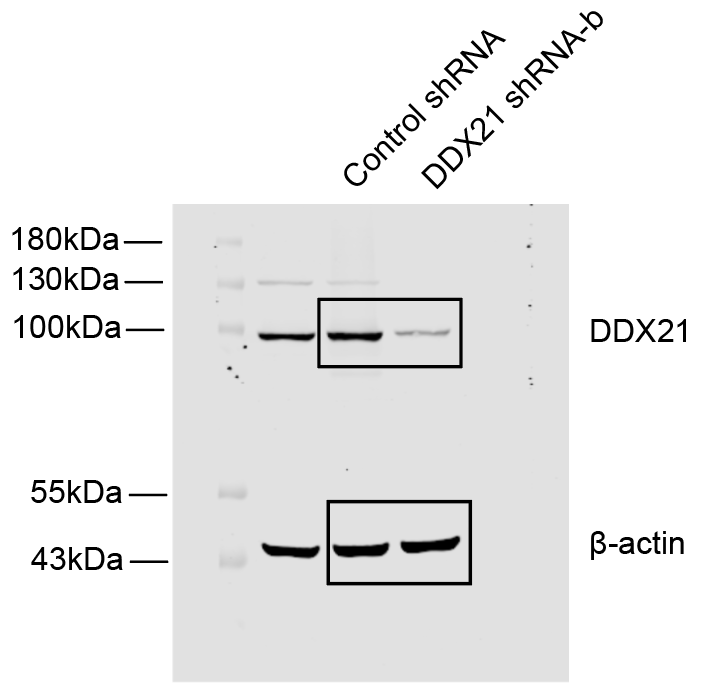


Supplementary Figure S3.A


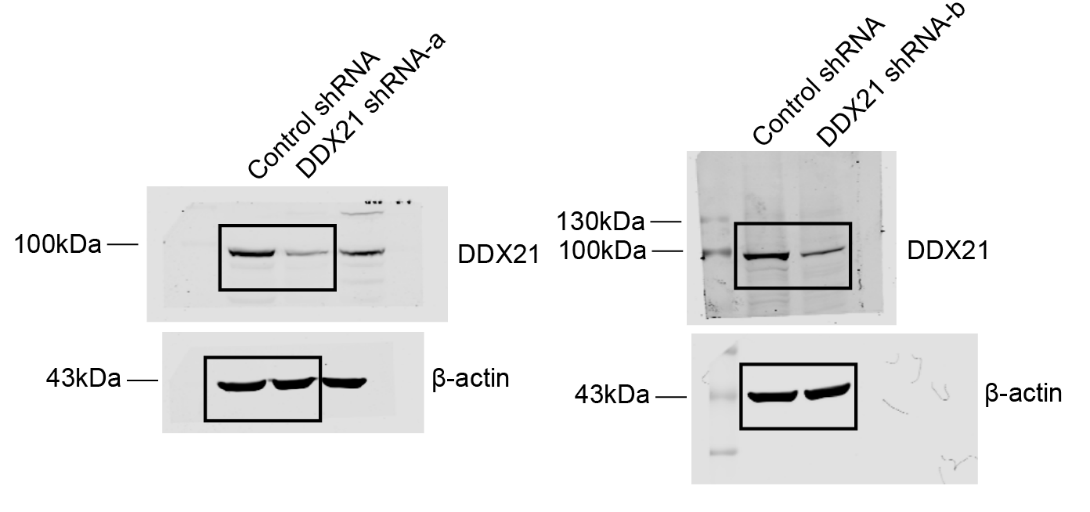


Supplementary Figure S5.A


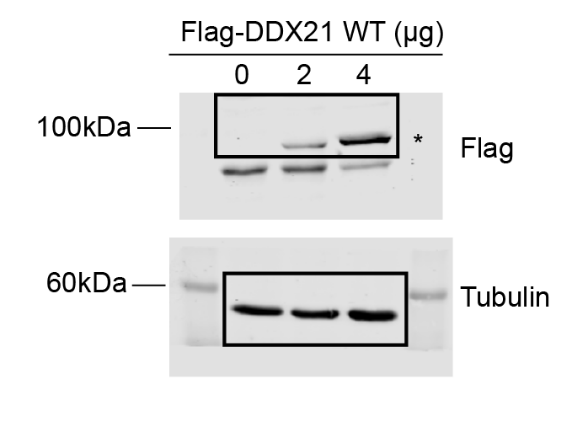


Supplementary Figure S6.A


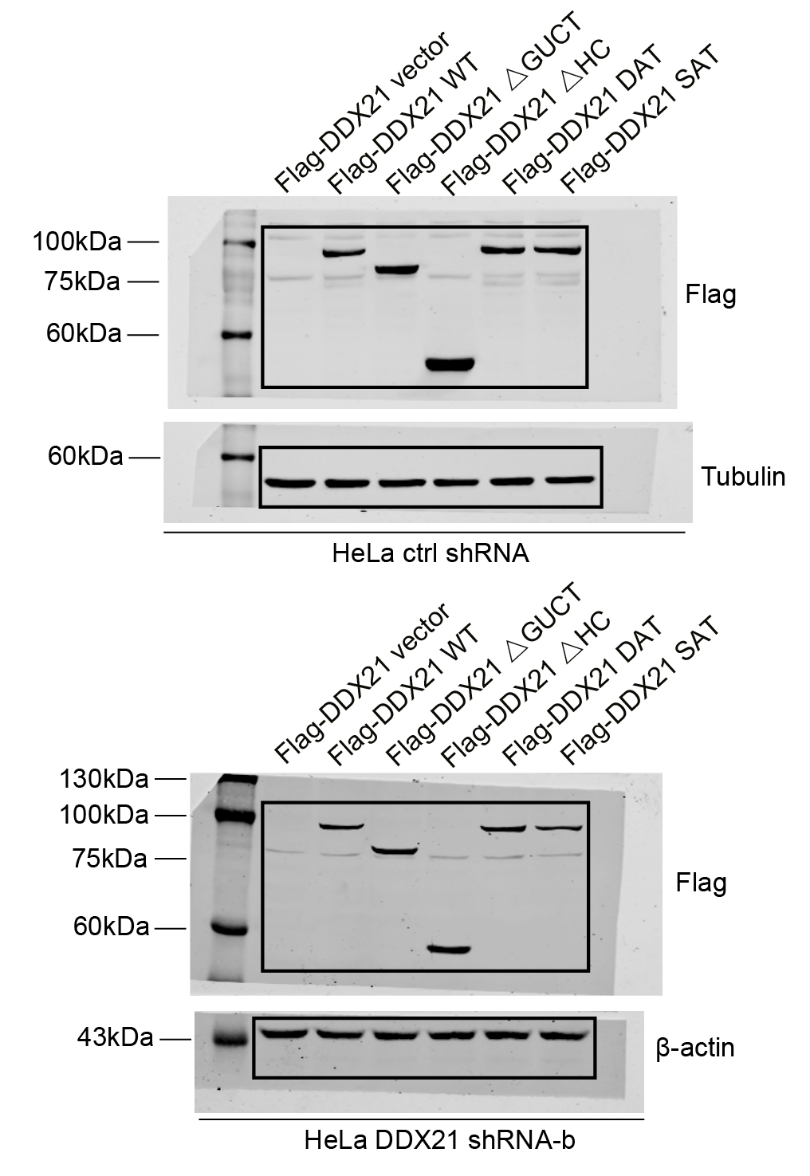


Supplementary Figure S9.A


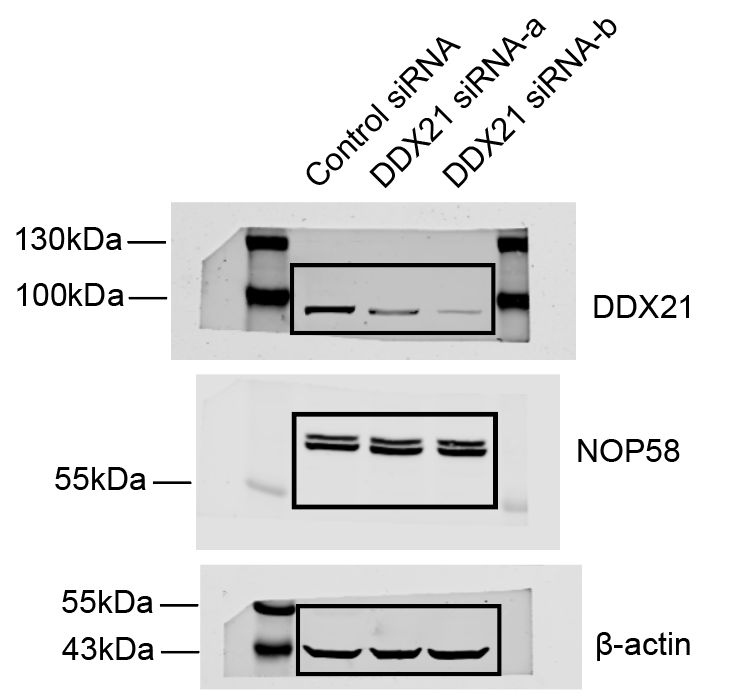


Supplementary Figure S10.D


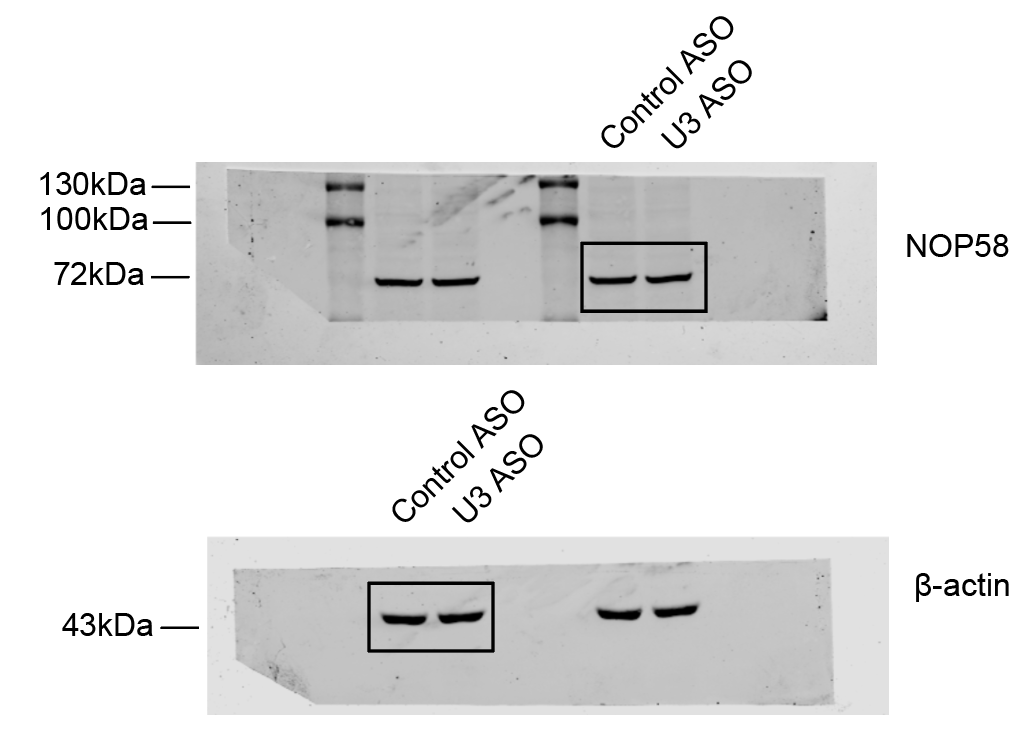


Supplementary Figure S12.B


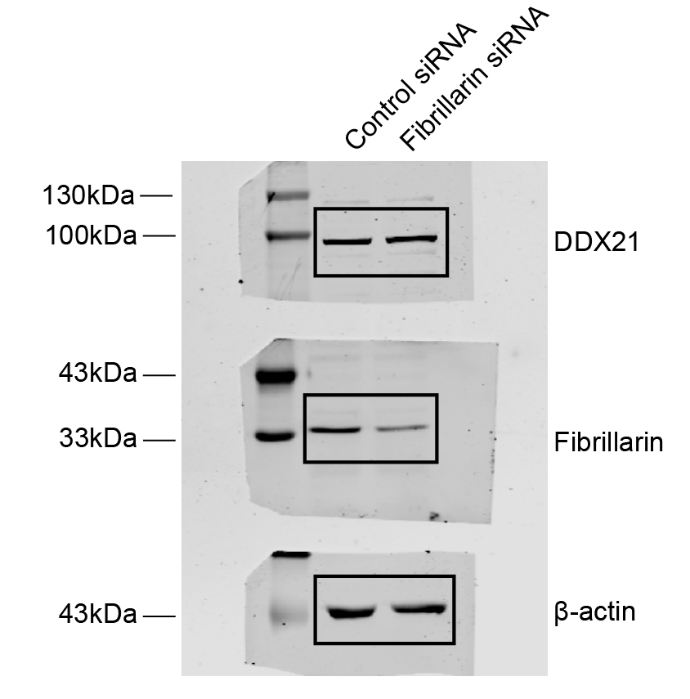

Supplement: Supplementary file 10 — Original Western blots [file 41419_2024_6725_MOESM10_ESM.docx]
